# Synthesis of Potential Haptens with Morphine Skeleton and Determination of Protonation Constants †

**DOI:** 10.3390/molecules25174009

**Published:** 2020-09-02

**Authors:** István Köteles, Károly Mazák, Gergő Tóth, Boglárka Tűz, Sándor Hosztafi

**Affiliations:** 1 Department of Pharmaceutical Chemistry, Semmelweis University, Hogyes Endre u. 9., H-1092 Budapest, Hungary; mazak.karoly@pharma.semmelweis-univ.hu (K.M.); toth.gergo@pharma.semmelweis-univ.hu (G.T.); tuz.boglarka@gmail.com (B.T.); hosztafi.sandor@pharma.semmelweis-univ.hu (S.H.); 2 Department of Plant Anatomy, Institute of Biology, Eötvös Loránd University, Pázmány Péter sétány 1/C, H-1117 Budapest, Hungary

**Keywords:** hapten, vaccine, immunotherapy, *N*-demethylation, nor-compounds, morphine skeleton, acid-base properties, protonation state, microspeciation

## Abstract

Vaccination could be a promising alternative warfare against drug addiction and abuse. For this purpose, so-called haptens can be used. These molecules alone do not induce the activation of the immune system, this occurs only when they are attached to an immunogenic carrier protein. Hence obtaining a free amino or carboxylic group during the structural transformation is an important part of the synthesis. Namely, these groups can be used to form the requisite peptide bond between the hapten and the carrier protein. Focusing on this basic principle, six nor-morphine compounds were treated with ethyl acrylate and ethyl bromoacetate, while the prepared esters were hydrolyzed to obtain the *N*-carboxymethyl- and *N*-carboxyethyl-normorphine derivatives which are considered as potential haptens. The next step was the coupling phase with glycine ethyl ester, but the reactions did not work or the work-up process was not accomplishable. As an alternative route, the normorphine-compounds were *N*-alkylated with *N*-(chloroacetyl)glycine ethyl ester. These products were hydrolyzed in alkaline media and after the work-up process all of the derivatives contained the free carboxylic group of the glycine side chain. The acid-base properties of these molecules are characterized in detail. In the *N*-carboxyalkyl derivatives, the basicity of the amino and phenolate site is within an order of magnitude. In the glycine derivatives the basicity of the amino group is significantly decreased compared to the parent compounds (i.e., morphine, oxymorphone) because of the electron withdrawing amide group. The protonation state of the carboxylate group significantly influences the basicity of the amino group. All of the glycine ester and the glycine carboxylic acid derivatives are currently under biological tests.

## 1. Introduction

Drug abuse is a worldwide problem. Even today, one of the most popular illegal substances is morphine and its derivative heroin, besides cocaine and marijuana. These drugs have a very serious potential to cause damage not just to the individual but to the whole society as well [1,2].

To help the drug addicts—and now let us just focus on the opiate users—there are few currently available clinical possibilities. The most common way is methadone therapy. Methadone is a potent opioid analgesic with longer duration of action compared to heroin or morphine. It can be a substitute for heroin and after withdrawal, the physical signs develop slower and seem to be less severe than those after heroin withdrawal. These properties are the basis for the detoxification of heroin addicts and methadone maintenance therapy, a long-term medication assisted treatment for opiate addiction. Theoretically this protocol works fine but in practice it can be easily spoiled. Namely, if the patient uses the illegal substance during the therapy all of the results that have been achieved so far are just a waste of time, money and energy. Not even to mention that the level of tolerance decreases as well and this can cause the death of the patient too. It is also a problem that relapses are quite common after the therapy.

For this problem an alternative solution can be vaccination against these drugs [3]. Drugs of abuse are small molecules that typically do not induce an antibody response following administration. To induce antibodies against these kinds of molecules, structural changes have to be made to obtain so called “haptens”. The hapten must be coupled to immunogenic proteins, called “carriers”. These connected derivatives are typically drug-linker adducts, in which the linker has a terminal functional group (i.e., carboxylic acid or aliphatic amine) that forms a covalent bond with the carrier. The efficacy of these conjugate vaccines depends on several factors including hapten design, coupling strategy, hapten density, carrier protein selection, and vaccine adjuvant [4,5].

Different opportunities are possible to functionalize the morphine structure: (i) *O*-alkylation the C3 phenolic hydroxyl group; (ii) esterification of the C6 hydroxyl group or oxime formation of the C6 carbonyl group; (iii) *N*-alkylation of the nor-compounds.

**Figure 1:**
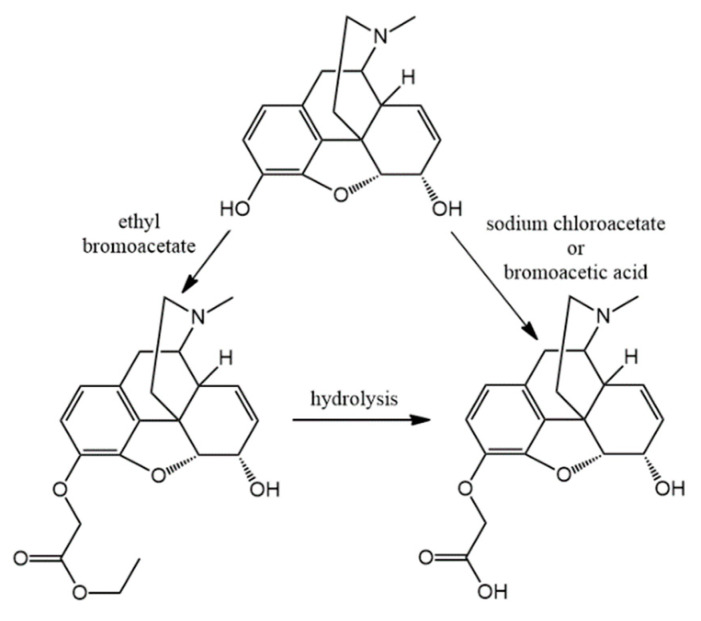
C3-hapten synthesis.

Spector and Parker reported the synthesis of the first morphine hapten which was a 3-*O*-alkylated compound [6,7] (Figure 1). Morphine was converted to 3-*O*-carboxymethylmorphine by reaction of the base with sodium chloroacetate in ethanol and the 3-*O*-carboxymethylmorphine was coupled to bovine serum albumin (BSA) with 1-ethyl-3(dimethylaminopropyl)-carbodiimide. This conjugate has immunogenic properties and it was suitable for the quantitative determination of morphine in serum by radioimmunoassay. Rubinstein and Ullman also prepared a 3-*O*-carboxymethylmorphine-BSA conjugate, and the free COOH group was activated by preparing the mixed anhydride with isobutyl chloroformate [8].

Buechler prepared 3-*O*-carboxymethylmorphine by refluxing of morphine base with bromoacetic acid in ethanol [9]. Heimann et al. converted morphine C3 potassium salt in ethanol and the solution was treated with ethyl bromoacetate. 3-Ethoxycarbonylmethyl-morphine was hydrogenated in the presence of Pd-C catalyst, and then 3-ethoxycarbonylmethyl-dihydromorphine [10] was hydrolyzed.

**Figure 2:**
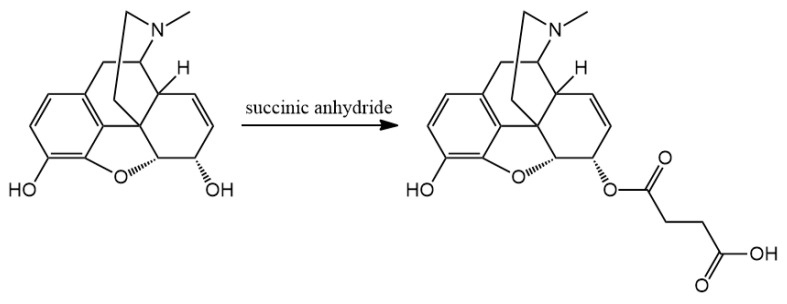
C6 ester-hapten synthesis.

For a C6 position modification, Wainer et al. prepared morphine-6-hemisuccinate ester by the reaction of morphine base with succinic anhydride [11,12,13,14] (Figure 2). This compound was simultaneously synthesized by Simon et al. [15]. In 1974 Bonese et al. used BSA conjugated morphine-6-hemisuccinate for the first time to vaccinate heroin dependent Rhesus monkeys [16]. After immunization, heroin self-administration in monkeys was blocked, and the antibody induced blockade was shown to be dose-dependent.

**Figure 3:**
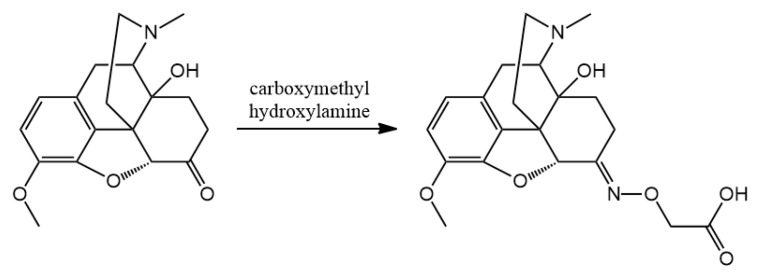
C6 oxime-hapten synthesis.

Pravetoni et al. developed haptens for vaccination of oxycodone and dihydrocodeinone [17,18,19]. These ketones were functionalized in position C6 (Figure 3) and C8. The reactions of oxycodone and dihydrocodeinone with carboxymethyl hydroxylamine led to C6 *O*-substituted oxime derivatives. These haptens were conjugated with BSA.

**Figure 4:**
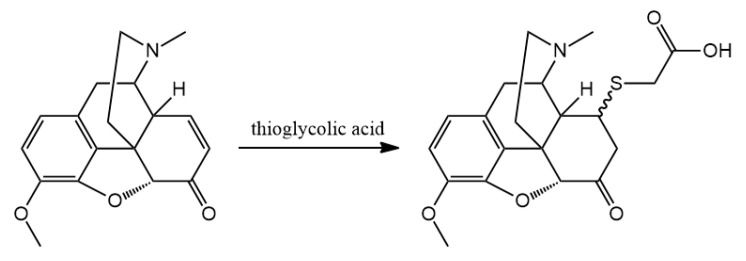
C8-hapten synthesis.

Codeinone was also transformed to a C8 substituted hapten. In this case the addition of an SH-group of thioglycolic acid took place on the double bond (Figure 4). The step of conjugation can be carried out with BSA.

Because BSA and KLH (keyhole limpet hemocyanine) are not allowed in human vaccinology, Anton and Leff used tetanus toxoid to conjugate morphine-6-hemisuccinate [20].

**Figure 5:**
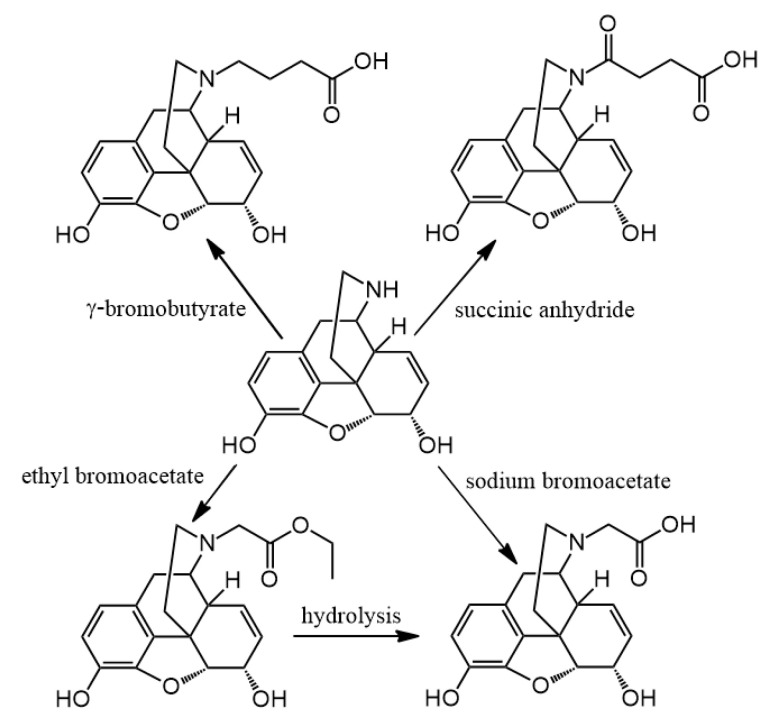
Bridge *N*-hapten synthesis.

Another way to design morphine haptens is to attach a COOH group, via a CH_2_ spacer, on the nitrogen atom of normorphinans and the carrier protein is conjugated to the nitrogen atom (Figure 5). Schneider converted normorphine with sodium bromoacetate to *N*-carboxymethyl-normorphine [21].

Gintzler et al. developed a radioimmunoassay method for the simultaneous determination of morphine and codeine. Normorphine was transformed with ethyl bromoacetate and the product *N*-ethoxycarbonylmethyl-normorphine (morphinan-17-acetic acid-7,8-didehydro-4,5-epoxi-3,6-dihydroxy-ethyl ester) was then hydrolyzed [22]. The product of the hydrolysis was coupled to BSA.

Findlay et al. used ethyl γ-bromobutyrate to *N*-alkylate normorphine and norcodeine in order to utilize a longer spacer [23]. The process was similar to Gintzler’s method. Herndon et al. also reported the synthesis of *N*-ethoxycarbonylmethyl-normorphine in the reaction of normorphine with ethyl bromoacetate. The ester was purified by silica gel column chromatography and hydrolysis of the ester resulted in *N*-carboxymethyl-normorphine [24].

Morris et al. prepared *N*-succinic-normorphine by carbodiimide coupling of normorphine with succinic acid [25]. This compound however did not contain basic nitrogen, after coupling to BSA yielded an immunoconjugate which was produced antisera after animal immunizations.

**Figure 6:**
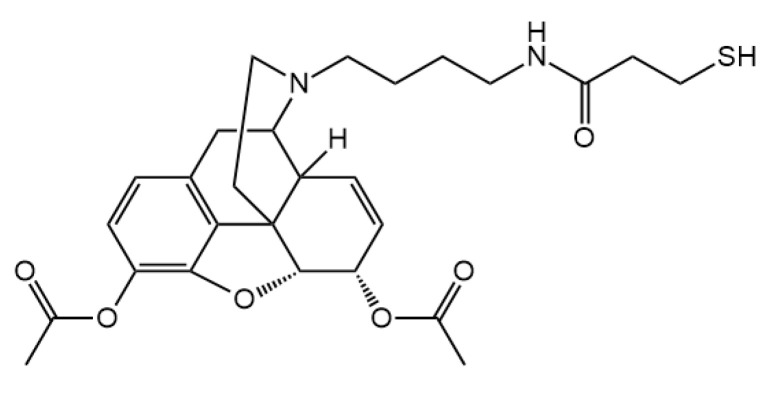
HerHap.

Stowe et al. in 2011 synthesized several haptens for the immunization of heroin. These molecules were conjugated to carrier proteins to receive heroin-vaccines [26,27]. 3,6-Diacetylnormorphine was treated with *N*-Boc-δ-aminobutanal and sodium triacetoxyborohydride to accomplish reductive amination. After removing the protecting group with trifluoroacetic acid (TFA) the free amino group was coupled with β-tritylmercaptopropionic acid *N*-hydroxysuccinimide active ester. Removing the trityl protecting group with TFA liberated the mercapto group (HerHap Figure 6) making it possible for further conjugation.

Recently a new type of functionalization of the aromatic ring has been elaborated, exchanging the phenolic hydroxyl group with an isostere amino group in order to connect function groups.

**Figure 7:**
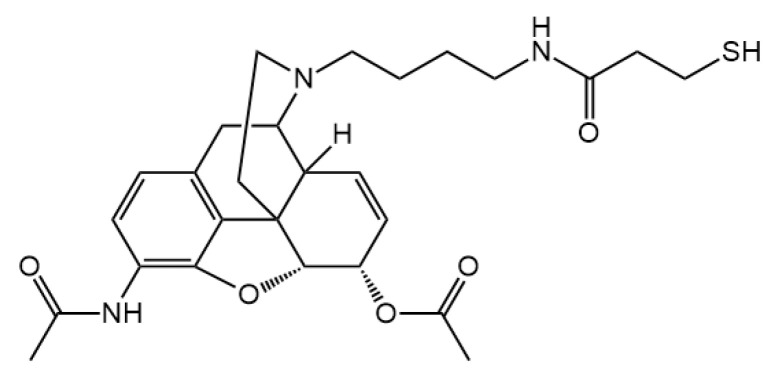
3-acetamido heroin hapten.

Bremer and Janda used 3-dezoxy-3-aminomorphine as the starting material for the synthesis of the heroin hapten [28]. After acetylation in the C6 position, this compound was *N*-demethylated and then *N*-alkylated to obtain the *N*-(δ-aminobutyl)-derivative. Because this compound contained an amide group in the C3 position instead of an ester, it was more stable against hydrolysis (Figure 7). It is a key factor of hapten development as the heroin-hapten is not stable: at pH = 7.4 and at room temperature, the shelf half-life is only 97 h. Because of this, the immunoconjugate practically has to be used right after the preparation.

**Figure 8:**
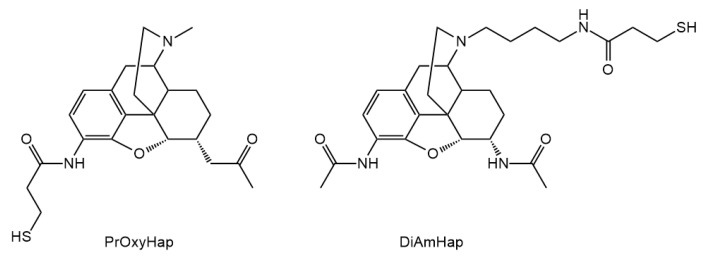
PrOxyHap and DiAmHap.

Li et al. reported the synthesis of similar hapten molecules and these were conjugated to tetanus toxoid [29]. The PrOxyHap (C3 hapten) contains a C3 aminogroup acylated with β-SH-propionic acid and a 2-oxopropyl moiety at position C6 and DiAmHap (*N*-bridge hapten), which contains acetamido groups at positions C3 and C6 as well as a δ-aminobutyl group on nitrogen (Figure 8).

These haptens were conjugated with tetanus toxoid. Mice were immunized and the antibody titer levels showed the following result: DiAmHap > 6-PrOxyHap. The latter caused the inhibition of the antinociceptive effects of heroin, morphine and 6-*O*-acetylmorphine in the animals, facillitated by the antigens.

**Figure 9:**
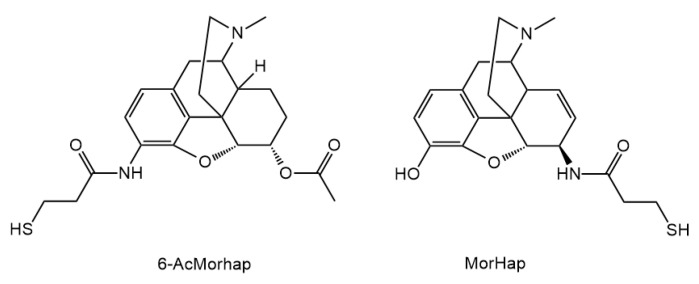
6-AcMorHap and MorHap.

Matyas et al. designed further heroin-like haptens [30]: they have synthesized the previously published HerHap (*N*-bridge hapten) by Stowe et al.; 6-AcMorHap (C3 hapten) is a 6-*O*-acetyl-morphine derivative that contains amino group in position C3 and this group is acylated with β-mercaptopropionic acid and MorHap (C6 hapten) is a derivative of 6-β-amino-6-desoxymorphine (Figure 9).

These haptens were conjugated to tetanus toxoid and they have produced high titers of antibodies in mice in the following order: MorHap > HerHap >> 6-AcMorHap.

The DiAmHap contains an isosteric acetamido group instead of an ester. Because of this only one type of antibody is induced that is reactive with the C3 and C6 acetyl-groups of heroin. In the case of the ester derivative, a heterogeneous population of antibodies was induced, because of the different structures of the hydrolyzed metabolites.

It is evident from the afore-mentioned survey, that the structure of the opiate skeleton can significantly influence the immunization properties of carrier protein coupled haptens. The purpose of this work was to synthesize *N*-substituted-4,5-epoxynormorphinans, carrying a free carboxylic group. We planned to examine the reactions of normorphinans with bromoacetic acid ethyl ester and with acrylic acid ethyl ester. The hydrolysis yields haptens with free COOH groups which can be connected with CH_2_ spacers. These compounds can be considered as morphinan skeleton *N*-substituted glycines and morphinan skeleton *N*-substituted β-amino propionic acids. We designed—for studying—the coupling of these carboxylic acid containing haptens with amino acid esters in order to model the coupling reactions of the hapten molecule with carrier proteins which contain free amino groups. For these novel compounds a detailed NMR analysis was planned.

## 2. Results and Discussion

### 2.1. Hapten Synthesis

Hapten molecules were designed with a morphine skeleton which contained free carboxylic groups on the nitrogen substituent connected with CH_2_ spacers to the nitrogen. Morphine, dihydromorphine, codeine, dihydrocodeine, oxycodone and oxymorphone were selected as model compounds and the syntheses of these hapten type derivatives included *N*-demethylation and *N*-alkylation reactions.

**Figure 10:**
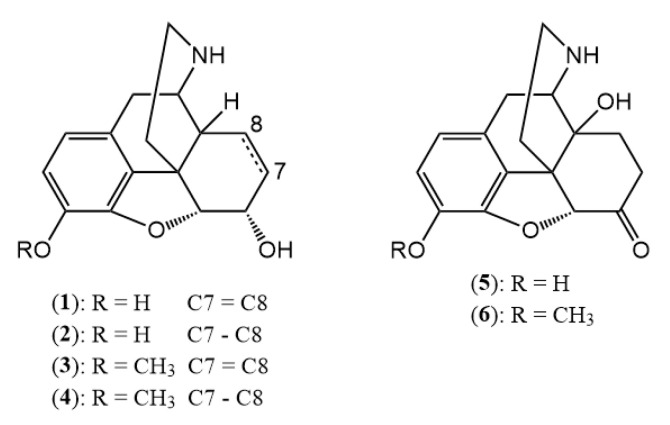
Normorphine derivatives.

For this purpose, the desired normorphine derivatives (normorphine (**1**), dihydronormorphine (**2**), norcodeine (**3**), dihydronorcodeine (**4**), noroxymorphone (**5**) and noroxycodone (**6**) (Figure 10)) can be obtained by the *N*-demethylation of the starting molecules [31,32,33,34,35,36,37].

**Scheme 1:**
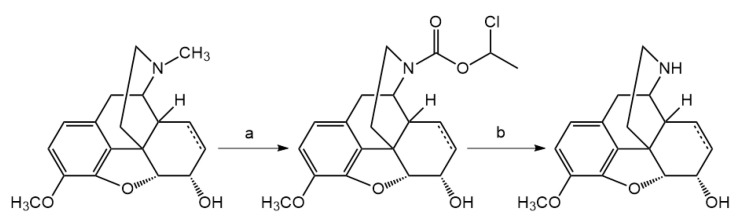
*N*-demethylation of (dihydro)codeine: (**a**) α-chloro-ethyl chloroformate, 1,2-dichloroethane; (**b**) methanol, heating.

Codeine or dihydrocodeine can be *N*-demethylated with α-chloro-ethyl chloroformate in 1,2-dichloroethane solvent and the intermediate carbamate was heated with methanol to yield the hydrochloride salt of norcodeine (dihydronorcodeine) (Scheme 1).

**Scheme 2:**
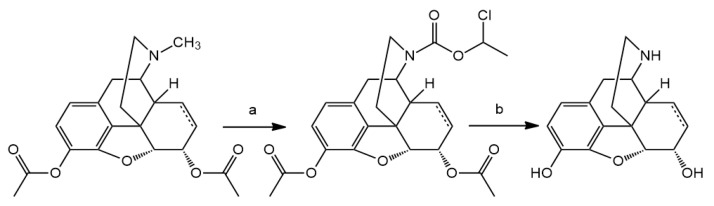
*N*-demethylation of (dihydro)morphine: (**a**) α-chloro-ethyl chloroformate, 1,2-dichloroethane; (**b**) methanol, heating, acid hydrolysis.

For the preparation of (dihydro)normorphine 3,6-diacetyl (dihydro)morphine was treated with α-chloro-ethyl chloroformate and after cleavage of the carbamate 3,6-diacetyl (dihydro)normorphine hydrochloride, salt was obtained. Acid hydrolysis of 3,6-diacetyl (dihydro)normorphine yielded (dihydro)normorphine (Scheme 2).

**Scheme 3:**
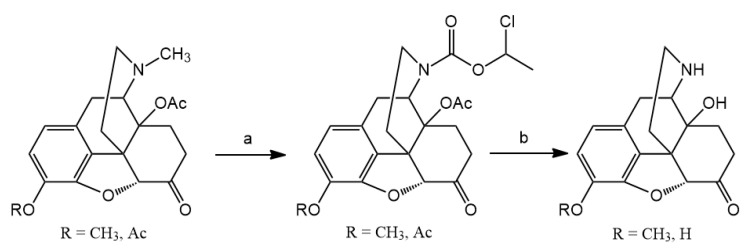
*N*-demethylation of oxymorphone and oxycodone: (**a**) α-chloro-ethyl chloroformate, 1,2-dichloroethane; (**b**) methanol, heating, acid hydrolysis.

3,14-di-*O*-Acetyloxymorphone and 14-*O*-acetyloxycodone were *N*-demethylated by the above procedure, and these reactions resulted in 3,14-di-*O*-acetylnoroxymorphone hydrochloride and 14-*O*-acetylnoroxycodone hydrochloride. After acid hydrolysis (10% HCl, 6h reflux) noroxymorphone and noroxycodone were isolated in high yields (Scheme 3).

The next step is the *N*-alkylation of the norcompounds. Depending on the linker chain size different methods were selected.

**Scheme 4:**
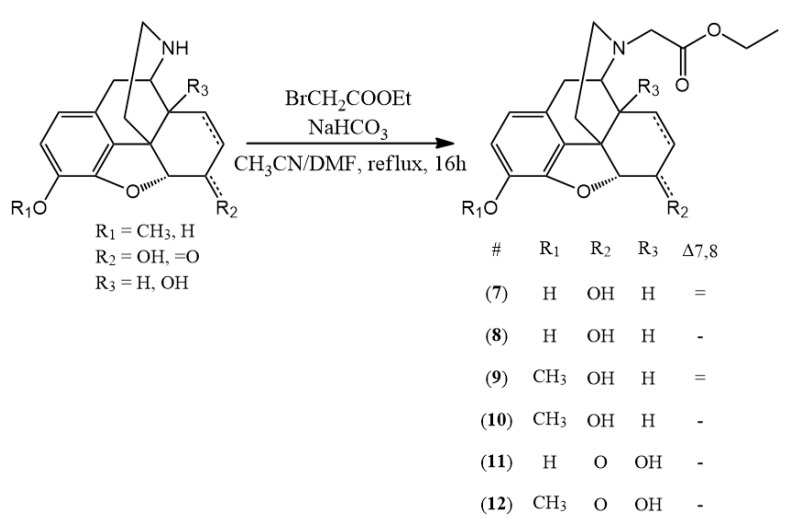
*N*-alkylation of nor-compounds: ethyl bromoacetate, sodium hydrogen carbonate, acetonitrile or dimethyl formamide, refl. 16 h.

In the case of the methylene bridge, ethyl bromoacetate was used (Scheme 4) and the starting nor-compound was dissolved in dimethyl formamide (DMF) or acetonitrile in the presence of sodium hydrogen carbonate, the reaction mixture was heated under reflux for 16 h. The conversion of the reaction was monitored by thin layer chromatography (TLC). In this reaction the *N*-carboxymethyl-nor-compound ethyl esters were obtained.

**Scheme 5:**
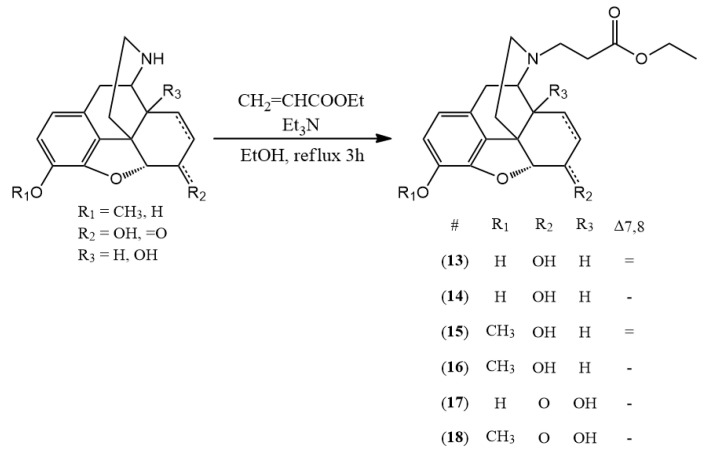
*N*-alkylation of nor-compounds: ethyl acrylate, triethylamine, ethanol, refl. 3 h.

To synthesize compounds with an ethylene linker, the reaction of ethyl acrylate with 4,5-epoxynormorphinans was studied (Scheme 5). The reaction was performed in ethanol and in the presence of triethylamine the mixture was heated under reflux for 3 h. The conversion was always complete and the products *N*-carboxyethyl-nor-compound ethyl esters were isolated in high yields. (In case of normorphine: morphinan-17β-propionic acid-7,8-didehydro-4,5-epoxi-3,6-dihydroxy-ethyl ester.)

**Scheme 6:**
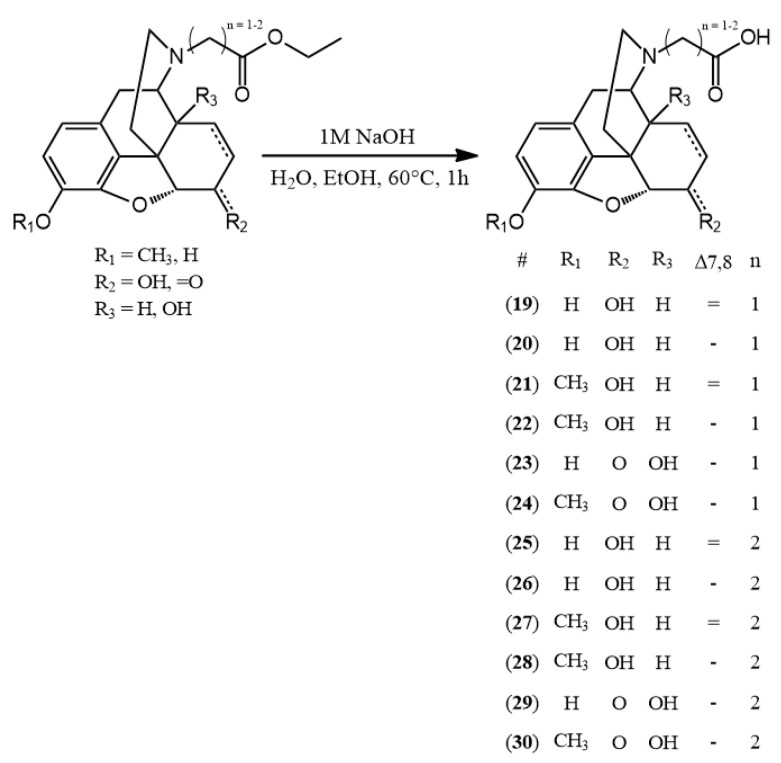
Hydrolysis of esters: 1 M NaOH, ethanol, water, heating, 1 h.

Then, the hydrolysis of the aforementioned esters (Scheme 6) with sodium hydroxide in ethanol at 60 °C, was studied. TLC was used to monitor the reaction. After complete conversion, the pH was adjusted to 3–4 and the mixture was evaporated till dry, to get the hydrochloride salt.

**Scheme 7:**
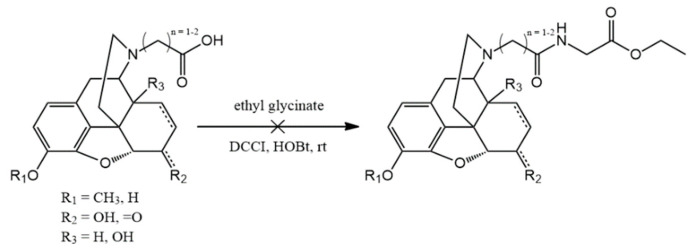
Attempted amino acid coupling: glycine ethyl ester, *N*,*N*′-dicyclohexyl carbodiimide (DCCI) or 1-ethyl-3-(3-dimethylaminopropyl)-carbodiimide (EDAC), 1-hydroxybenzotriazole (HOBt), water, room temperature.

In the next step, the reaction of the free carboxylic group containing molecules with glycine ethyl ester was attempted (Scheme 7). Reagents which are common in peptide synthesis were used like *N*,*N*′-dicyclohexyl carbodiimide (DCCI) or 1-ethyl-3-(3-dimethylaminopropyl)-carbodiimide (EDAC) and 1-hydroxybenzotriazole (HOBt). Unfortunately, these couplings gave very low yields and the mixtures did not result in the expected compounds. The *N*-acylated glycine esters were not able to be isolated. In order to prepare these target compounds, another synthesis route was selected.

**Scheme 8:**
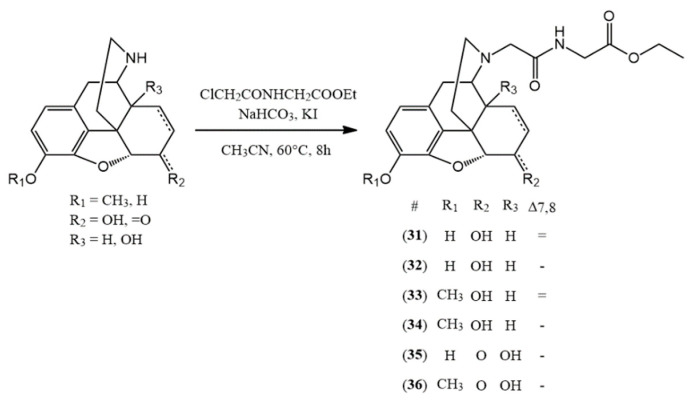
Amino acid connected hapten: *N*-chloroacetlyglycine ethyl ester, sodium hydrogen carbonate, potassium iodide, acetonitrile, 60 °C, 8 h.

In the cases of the methylene bridge-containing compounds, the retro-synthetic analysis revealed that the linker between the nor-compounds and the glycine ethyl ester can be derivatized with chloroacetyl chloride and *N*-alkylation with *N*-(chloroacetyl)glycine ethyl ester, resulting in the target compounds (Scheme 8).

**Scheme 9:**
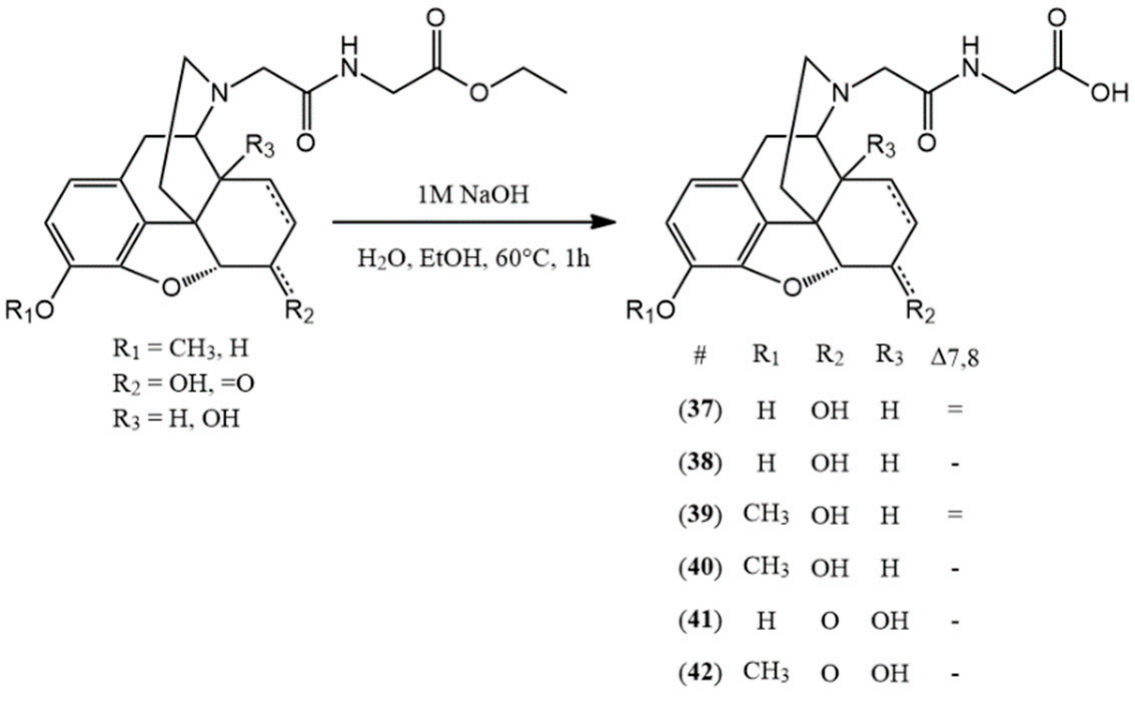
Hydrolysis of *N*-acetylglycine esters: 1 M NaOH, ethanol, water, heating, 1 h.

The nor-compound was treated with the *N*-(chloroacetyl)glycine ethyl ester in acetonitrile in the presence of potassium iodide and sodium hydrogen carbonate. The mixture was heated and stirred at 60 °C until conversion was complete. The products were isolated by the usual work-up and purification of the compounds was performed by column chromatography. These esters were hydrolyzed using the aforementioned reaction conditions (Scheme 9).

Structures of all the above mentioned compounds were confirmed with 1D and 2D NMR as well as HR-MS measurements.

### 2.2. Protonation Constants of the N-Acetylglycine Opioid Compounds

The most important physicochemical properties influencing the pharmacokinetic behavior of drugs and biomolecules are the acid-base properties, lipophilicity, solubility and permeability, all related to passive absorption [38].

The acid-base character determines the ionization state of a molecule in a solution of a particular pH. Consequently, all pharmacokinetic properties, namely absorption, distribution, metabolism, excretion and toxicity (ADMET) are influenced by the ionization state under varying pH conditions [39].

*N*-acetylglycine opioid compounds can have up to three basic functional groups, namely an amino and a phenolate on the main opioid skeleton, and in the glycine side chain a carboxylate group. Such compounds are tribasic and can be characterized by three protonation constants, *K*_1_, *K*_2_ and *K*_3_. Compounds with a methoxy group in place of the phenolate are dibasic. Esterification of the carboxylic site further reduces the number of basic groups, so the esters of codeine derivatives contain just an amino group as a basic site.

After the synthesis of the new *N*-acetylglycine opioid compounds, their protonation macroconstants were also determined to characterize their acid-base properties. The ionization state of molecules under the diverse conditions of pH in the various parts of the body influences all their pharmacokinetic properties during absorption, distribution, metabolism and excretion. The binding to target molecules (the pharmacodynamic activity), occurs at a definite ionization state. Acid-base properties play a significant part in the formulation of drug substances for both oral dosage and intravenous forms as well [40].

We have recently published a review on the site-specific acid-base properties of morphine and related compounds [41], where it was shown that the basicity of the amino and phenolate site is usually comparable, with both of them protonating in slightly alkaline solutions. Protonation constants *K*_1_ and *K*_2_ of the tribasic compounds describe the uptake of the first two protons on these functional groups. The basicity of the carboxylate site is smaller by several orders of magnitude, thus *K*_3_ values almost exclusively characterize the protonation of this site.

The main goal of the study of the protonation constants was to see how these newly synthesized side chains with different electron withdrawing groups (amide, carboxylic acid ester, carboxylate) influence the basicity of the amino group in the vicinity. A potentially smaller basicity of the amino group could change the relative concentration of the zwitterionic form compared to the parent compounds, influencing many pharmacokinetic properties.

pH-potentiometry is the standard method for the determination of protonation constants [38]. This technique was used to obtain the protonation constants of the compounds, with the exception of the rather small log *K*_3_ values where NMR-pH titrations were carried out using indicator molecules to show the exact pH values in highly acidic media.

Although these compounds contain up to twenty protons connected to carbon atoms, in the acidic pH region, the chemical shift of only the protons that are located close to the protonating carboxylate site changes significantly. The signals of the methylene and amide protons in the glycine side chain were followed, connected to carbon and nitrogen atoms, respectively. None of the other protons showed a significant change in their chemical shifts in acidic solutions.

The protonation constants are collected in Table 1.

The vicinity of the electron withdrawing amide group significantly decreases the basicity of the amino group, thus, in these opioid derivatives the basicity of the phenolate site is much larger than that of the amino site. Consequently, the *K*_1_ constant practically characterizes the basicity of the phenolate site, whereas *K*_2_ characterizes that of the amino site, when the phenolate already holds a proton. The *K*_3_ constant can be ordered to the carboxylate site when the other two sites are already protonated.

In the morphine-dihydromorphine pairs, hydrogenation of the C7-C8 double bond increases the electron density and thus, the basicity of both the phenolate and the amino sites. In the morphine-oxymorphone pairs the replacement of the C6 hydroxyl group by the electron withdrawing keto group, decreases the basicity of both the phenolate and the amino sites.

The higher amino basicity of the carboxylic acids compared to their ester derivatives can be interpreted by the fact that in the pH range of the amino protonation, the carboxyl groups are predominantly deprotonated, thus negatively charged. Hence they do not have a strong electron withdrawing effect, unlike the uncharged ester groups. The *K*_3_ values, just as the *K*_2_ values of the methoxy derivatives characterize the protonation of the carboxylate sites in acidic medium and are practically identical in all the investigated compounds. This observation can be explained by the fact that the carboxylate site lies several covalent bonds away from the opioid skeleton, so any changes introduced in that skeleton will have practically no effect on the carboxylate basicity.

**Figure 11:**
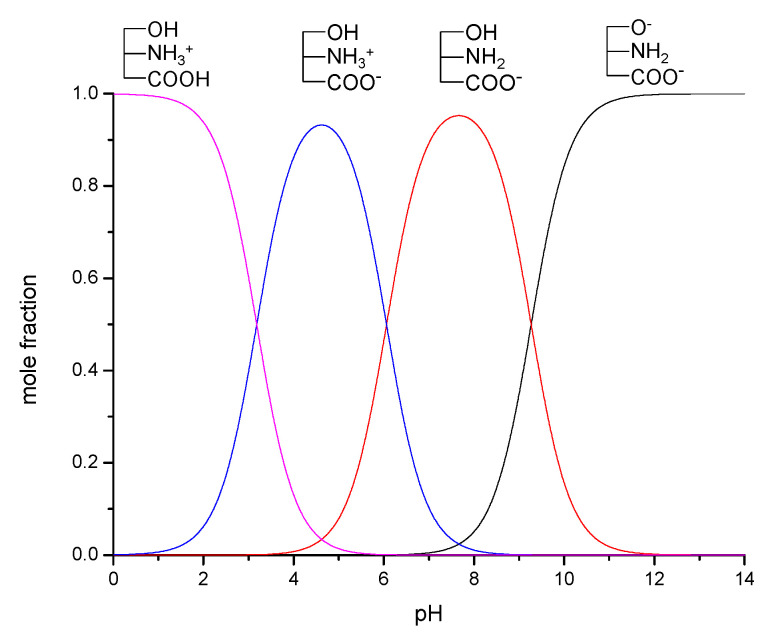
The species distribution diagram of *N*-acetylglycine normorphine.

The determination of protonation constants allows the construction of species distribution diagrams, shown here for *N*-acetylglycine normorphine (**37**), as example (Figure 11). The protonation state of each group can be seen above the curves.

The intersections of the species distribution curves show that in the pH range 6.06–9.27 this compound mainly exists in the anionic, in more alkaline solutions in the dianionic form. In slightly acidic pH values the zwitterionic form has the highest contribution to the mole fraction, whereas in more acidic solutions the cationic form is dominant.

### 2.3. Protonation Constants of the N-Carboxyalkyl Opioid Compounds

The side-chain of these molecules does not have an electron withdrawing amide group, thus, the basicity of the amino and phenolate sites becomes comparable. In such cases protonation microconstants are needed to describe the basicity of the variously protonated forms [39].

**Figure 12:**
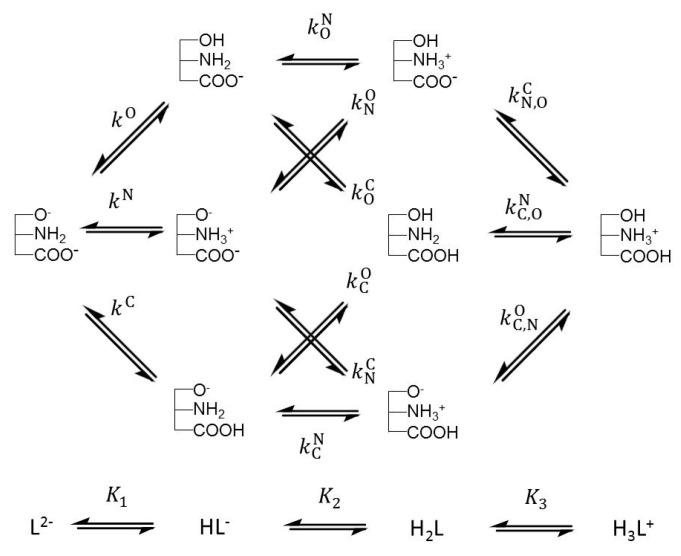
The protonation scheme of tribasic *N*-carboxyalkyl opioid compounds.

Tribasic *N*-carboxyalkyl opioid compounds exist in solutions in eight microscopic protonation forms (Figure 12) and twelve microconstants are needed to describe their protonation microequilibria.

*K*_1_, *K*_2_ and *K*_3_ are the stepwise macroconstants, small case *k* stands for the microconstants, indices C, N and O designate the carbon, nitrogen and oxygen atoms in the carboxylate, amino and phenolate groups, respectively. Superscripts of microconstants indicate the group protonating in the given microequilibrium protonation process, whereas the subscript (if any) stands for the group already holding proton during the process [39].

Some of the relationships between the micro and macroconstants of tribasic *N*-carboxyalkyl opioid compounds are as follows:

(1) $$\beta_{1}=K_{1}=k^{N}+k^{O}+k^{C}$$

(2) $$\beta_{3}=K_{1}K_{2}K_{3}=k^{O}k_{O}^{N}k_{N,O}^{C}=k^{C}k_{C}^{N}k_{C,N}^{O}=\cdots$$

**Figure 13:**
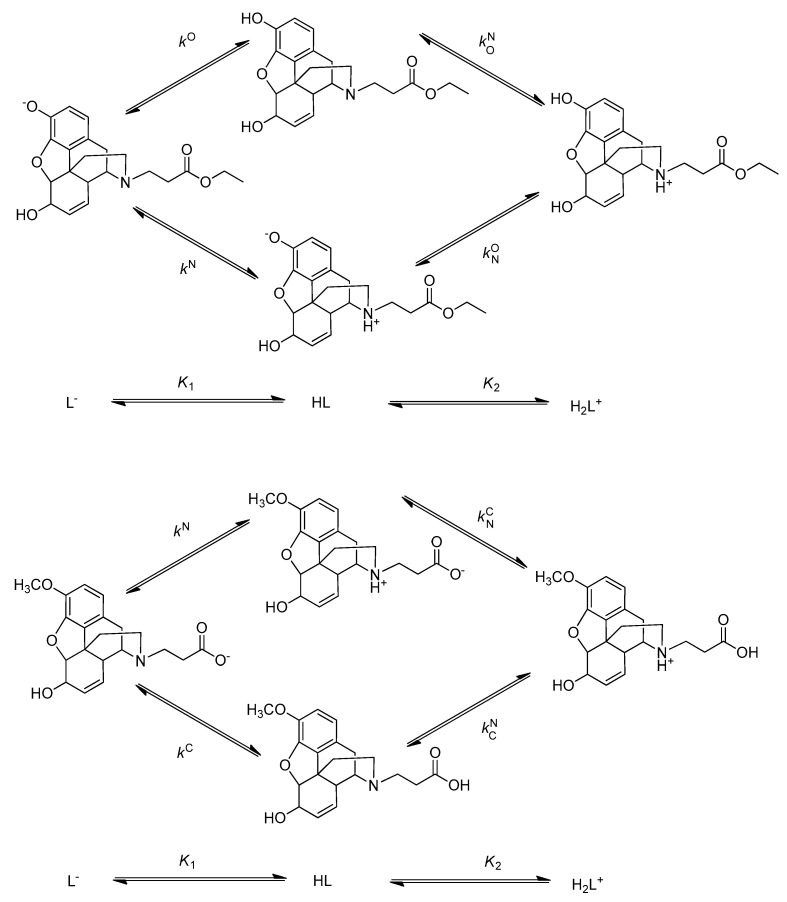
The microscopic protonation forms and microspecies of *N*-carboxyethyl-normorphine ethyl ester (**13**) and *N*-carboxyethyl-norcodeine (**27**).

*N*-carboxyalkyl opioid compounds with either a methoxy or an ester function are only dibasic, and exist in solutions in four microscopic protonation forms. Such ligands (abbreviated as L) carry one negative charge in alkaline solutions and upon the uptake of a proton, they will be transformed into either a non-charged or zwitterionic form. These two forms are protonation isomers, differing from each other only in the site of protonation. The uptake of a second proton produces a single cationic species. These four microspecies, alongside their respective protonation microconstants, are shown in Figure 13, exemplified by *N*-carboxyethyl-normorphine ethyl ester (**13**) and *N*-carboxyethyl-norcodeine (**27**).

#### 2.3.1. Determination of the Protonation Macroconstants

**Figure 14:**
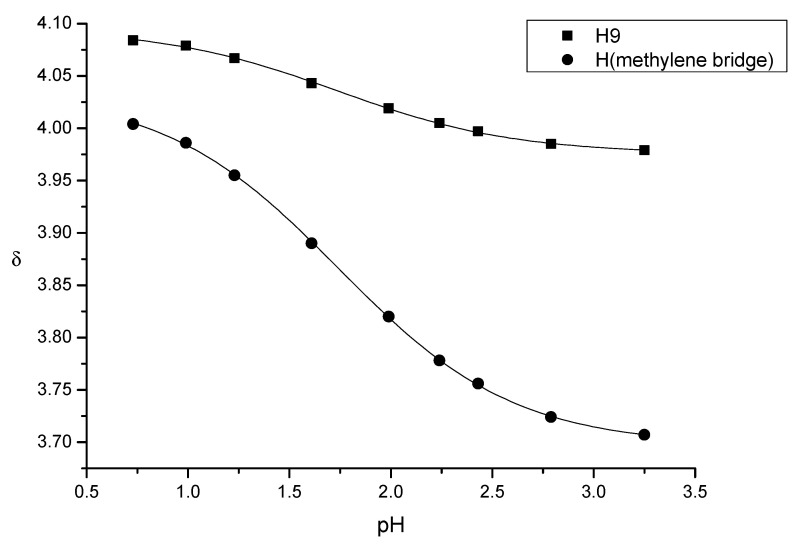
NMR-pH titration curves of the H9 and methylene bridge protons of *N*-carboxymethyl-noroxymorphone (**23**). Computer fits for log *K*_3_ are shown in solid lines.

For the determination of protonation macroconstants the same strategy was followed as outlined above for the *N*-acetylglycine opioid derivatives. pH-potentiometry was used to obtain the protonation macroconstants, with the exception of the rather small log *K*_3_ values, where NMR-pH titrations were carried out using indicator molecules. Typically, the doublets of the H9 proton and the signals of the protons in the ethylene or methylene bridge connecting the amino and carboxylate sites were followed, unless they were overlapped by other interfering signals. The NMR-pH titration curve of *N*-carboxymethyl-noroxymorphone (**23**) can be seen in Figure 14.

The protonation macroconstants are collected in Table 2.

#### 2.3.2. Determination of the Protonation Microconstants of the Dibasic Methoxy Derivatives

Dibasic methoxy derivatives contain an amino and a much less basic carboxylate group, thus the deductive method was chosen for the determination of microconstants, which requires an appropriate model compound to mimic the minor species [39]. The macroconstants of the methoxy ester derivatives were used as the $k_{C}^{N}$ microconstants of the dibasic methoxy derivatives. For dibasic compounds, in the knowledge of one microconstant and the two macroconstants, all other microconstants can be calculated (Table 3). Table 3 also contains the log*E*_C,N_ pair-interactivity parameter that characterizes how the protonation at one basic site decreases the basicity of the other site.

(3) $$\log E_{C,N}=\log k^{N}-\log k_{C}^{N}=\log k^{C}-\log k_{N}^{C}$$

#### 2.3.3. Determination of the Protonation Microconstants of Tribasic Compounds

In order to determine all the microconstants of the investigated tribasic compounds, the line of thought detailed below was followed. As already discussed, *K*_3_ is practically equal to $k_{N,O}^{C}$, since protonation along this arrow overwhelmingly predominates the $k_{C,N}^{O}$ and $k_{C,O}^{N}$ processes. The macroconstants of the methoxy ester derivatives can be inserted in place of the $k_{C,O}^{N}$ microconstants. With the help of the log*E*_C,N_ pair-interactivity parameter from Table 3, $\log k_{O}^{N}$ and $\log k_{O}^{C}$ can be obtained. $\log k_{C}^{O}$ can be calculated from the macroconstants of the ester derivatives using the value of $k_{C,O}^{N}$. *k*^C^ and *k*^O^ can be obtained from *β*_3_ using Equation (2). Once these microconstants are known, *k*^N^ can be calculated from Equation (1). All the other microconstants can be obtained from Equation (2) and the log*E*_C,N_ pair-interactivity parameter. From the microconstants, the log*E*_N,O_ and log*E*_C,O_ pair-interactivity parameters can also be calculated analogous to Equation (3). The results are displayed in Table 4.

#### 2.3.4. Determination of the Protonation Microconstants of the Dibasic Ester Derivatives

Dibasic ester derivatives contain an amino and phenolate group. As the protonation of the phenolate group cannot be selectively monitored by UV spectroscopy for morphine derivatives, the deductive method was chosen for the determination of microconstants. The macroconstant of the methoxy ester derivative is very close to the $k_{O}^{N}$ microconstant of the ester derivative, because this microconstant lies on the dominant protonation pathway. Thus, the microconstants of the minor protonation pathway can only be calculated with the help of the log*E*_N,O_ pair-interactivity parameter from Table 4. The results are displayed in Table 3.

#### 2.3.5. Discussion of the Protonation Constants of the N-Carboxyalkyl Opioid Compounds

The basicity of the amino and phenolate site is within an order of magnitude in each tribasic molecule. In the morphine-dihydromorphine pairs, hydrogenation of the C7-C8 double bond increases the electron density and thus the basicity of both the phenolate and the amino sites.

The higher amino basicity of the carboxylic acids compared to their ester derivatives can once again be interpreted by the fact that in the pH range of the amino protonation, the negatively charged carboxylate groups do not have a strong electron withdrawing effect, unlike the uncharged ester groups. On the other hand, the charge of the side chain of the amino group does not significantly influence the basicity of the far-away phenolate site, resulting in log*E_C_*_,O_ pair-interactivity parameters being close to zero. $k_{N,O}^{C}$ values that characterize the protonation of the carboxylate sites in acidic medium are much smaller for *N*-carboxymethyl than those of the *N*-carboxyethyl derivatives. This observation can be explained by the fact that the protonated, electron withdrawing amino group is closer to the carboxylate in the *N*-carboxymethyl derivatives, and is further manifested in much higher pair-interactivity parameters between these two groups.

**Figure 15:**
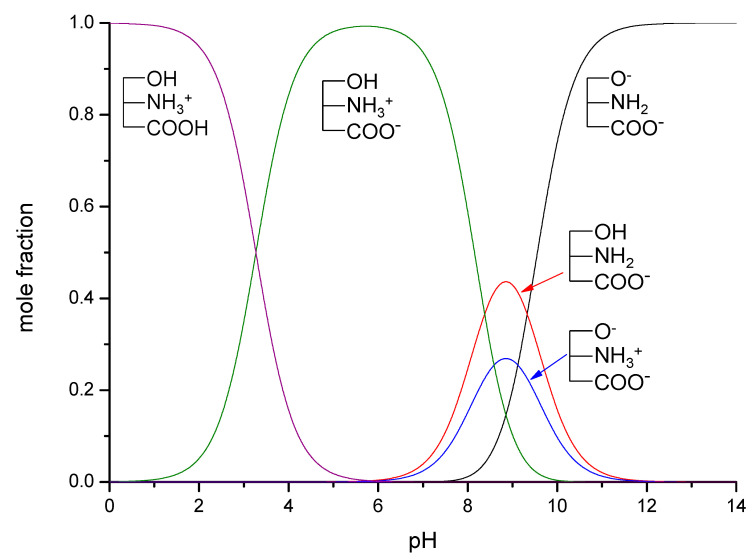
The microspecies distribution diagram of *N*-carboxyethyl-normorphine (**25**).

The concentration of the various microconstants can be shown in distribution diagrams, shown here for *N*-carboxyethyl-normorphine (**25**) (Figure 15).

The intersections of the species distribution curves show that in the pH range 3.27–8.38 this compound mainly exists in the zwitterionic form. In more acidic pH values, the cationic form has the highest contribution to the mole fraction, whereas in more alkaline solutions the anion protonated on the phenolate site, and finally the dianion is dominant.

## 3. Materials and Methods

### 3.1. General Information

The reagents and indicator molecules were purchased from Sigma-Aldrich (St. Louis, MO, USA) and Alfa Aesar (Haverhill, MA, USA) and used without further purification. Solvents were freshly distilled prior to use and were dried over anhydrous Na_2_SO_4_. ^1^H and ^13^C-NMR spectra were recorded on a 600-MHz Varian VNMRS spectrometer (Varian, Inc., NMR Systems, Palo Alto, CA, USA, Varian is now part of Agilent Technologies) in DMSO-*d*_6_ or D_2_O solutions; δ is given in ppm relative to tetramethylsilane (TMS) as internal standard. ^1^H and ^13^C-NMR signals were assigned on the basis of one- and two-dimensional homo- and heteronuclear experiments (COSY, TOCSY, HMBC and HSQC). Melting points were taken on a Stuart SMP-3 apparatus (Global Science NZ Ltd., Auckland, New Zealand). The high-resolution accurate masses were determined with a Dionex Ultimate 3000 UHPLC system hyphenated with an Orbitrap Q Exactive Focus Mass Spectrometer equipped with electrospray ionization (ESI) (Thermo Fischer Scientific, Waltham, MA, USA). Reaction progress was observed by thin-layer chromatography on commercial silica gel plates (Merck silica gel F254 on aluminum sheets, Darmstadt, Germany) using different mobile phases. For column chromatography, Kieselgel 60 (particle size 0.040–0.063 mm, ordered from VWR Chemicals, Radnor, PA, USA) was employed.

### 3.2. Hapten Synthesis

Altogether 36 compounds have been synthetized and most of them are new molecules, only *N*-carboxymethyl-normorphine ethyl ester, and *N*-carboxyethyl-norcodeine ethyl ester were previously synthesized [42,43]. The synthesis of nor-compounds (normorphine, dihydronormorphine, norcodeine, dihydronorcodeine, noroxymorphone and noroxycodone) by *N*-demethylation with α-chloroethyl chloroformate was accomplished. Nor-compounds were converted to *N*-carboxyethyl methyl derivatives with ethyl bromoacetate and nor-compounds were also converted to *N*-carboxyethyl ethyl derivatives with the reaction of ethyl acrylate. The *N*-substituted esters were hydrolyzed in order to obtain compounds with free carboxylic acid on nitrogen. The coupling of these free carboxylic groups with amino acid esters was attempted but these reactions were unsuccessful and the expected products were could not be isolated. Therefore, a new reaction was elaborated in order to obtain hapten coupled amino acid esters. The conversion of all nor-compounds was achieved by the reaction with *N*-chloroacetyl amino acid esters. Finally, the alkaline hydrolysis of these *N*-acetylglycine-normorphinan ethyl esters was achieved too.

#### 3.2.1. *N*-Demethylation Reactions

Codeine or dihydrocodeine (3 mmol) was dissolved in dried 1,2-dichloroethane (30 mL), then sodium hydrogen carbonate (0.84 g) was added to the solution. To this ice-cold mixture, α-chloroethyl chloroformate (10 mmol) was added dropwise and the reaction mixture was stirred at room temperature for 30 min., then heated at 90 °C overnight. The resulting suspension was cooled to room temperature and inorganic salts were filtered off. The filtrate was evaporated under reduced pressure and the residue was dissolved in methanol and the solution was heated at 60 °C for 6 h. Methanol was removed under reduced pressure to yield a crystalline solid, the hydrochloride salt of norcodeine or dihydronorcodeine. Free base was liberated with 10% sodium hydroxide (pH = 9) and was extracted with chloroform. The chloroform extract was dried (sodium sulfate) and evaporated to result in the nor-compound.

Norcodeine (**3**) yield: 87%, m.p.: 185 °C (acetone); ^1^H-NMR (600 MHz, DMSO-*d_6_*) δ 6.60 (d, *J* = 8.2 Hz, H-2), 6.44 (d, *J* = 8.2 Hz, H-1), 5.52 (dp, *J* = 9.7, 1.5 Hz, H-7), 5.21 (dt, *J* = 9.8, 2.8 Hz, H-8), 4.64 (dd, *J* = 5.9, 1.4 Hz, H-5), 4.09 (dh, *J* = 5.4, 2.6 Hz, H-6), 3.72 (s, OCH_3_), 3.46 (dd, *J* = 5.9, 3.3 Hz, H-9), 2.71 (m, H-10), 2.67 (m, H-16), 2.65 (m, H-10), 2.46 (q, *J* = 2.8 Hz, H-14), 1.82 (td, *J* = 12.2, 5.5 Hz, H-15), 1.59 (dt, *J* = 12.3, 2.4 Hz, H-15); ^13^C-NMR (150 MHz, DMSO-*d_6_*) δ 147.7 (C4), 141.6 (C3), 133.7 (C7), 131.8 (C12), 129.1 (C8), 128.3 (C11), 118.7 (C1), 113.6 (C2), 93.1 (C5), 66.8 (C6), 56.5 (OCH3), 51.5 (C9), 44.3 (C13), 41.1 (C14), 38.4 (C16), 36.9 (C15), 31.3 (C10)

Dihydronorcodeine (**4**) yield: 83%, m.p.: 194 °C (ethanol); ^1^H-NMR (600 MHz, DMSO-*d_6_*) δ 6.69 (d, *J* = 8.1 Hz, H-2), 6.53 (d, *J* = 8.1 Hz, H-1), 4.43 (d, *J* = 4.9 Hz, H-5), 3.81 (m, H-6), 3.76 (s, OCH_3_), 3.17 (s, H-9), 2.81 (dd, *J* = 18.1, 6.2 Hz, H-10), 2.61 (m, H-10), 2.59 (m, H-16), 2.54 (m, H-16), 2.06 (ddd, *J* = 11.8, 6.7, 2.8 Hz, H-14), 1.66 (td, *J* = 12.3, 5.2 Hz, H-15), 1.45 (m, H-15), 1.36 (m, H-8), 1.32 (m, H-7), 1.18 (m, H-7), 0.83 (tt, *J* = 11.9, 6.1 Hz, H-8); ^13^C-NMR (150 MHz, DMSO-*d_6_*) δ 147.5 (C4), 141.2 (C3), 130.9 (C12), 127.8 (C11), 118.5 (C1), 114.4 (C2), 91.1 (C5), 66.3 (C6), 56.7 (OCH_3_), 52.3 (C9), 43.0 (C13), 39.1 (C14), 38.4 (C16), 38.1 (C15), 30.5 (C10), 26.4 (C7), 20.2 (C8)

(Dihydro)normorphine was prepared by *N*-demethylation of diacetyl (dihydro)morphine by means of α-chloro-ethyl chloroformate using the above-mentioned procedure and the carbamate was treated with methanol at 60 °C to afford the diacetyl (dihydro)normorphine hydrochloride salt. (Dihydro)normorphine was obtained by hydrolysis of diacetyl (dihydro)normorphine × HCl in 5% HCl at 100 °C for 4 h. (Dihydro)normorphine base was precipitated with 25% ammonia solution (pH = 9–10) and filtered off.

Normorphine (**1**) yield: 84%, m.p.: 273–275 °C; ^1^H-NMR (600 MHz, DMSO-*d_6_*) δ 6.46 (d, *J* = 8.0 Hz, H-2), 6.35 (d, *J* = 8.1 Hz, H-1), 5.55 (dp, *J* = 9.9, 1.5 Hz, H-7), 5.20 (dt, *J* = 9.8, 2.8 Hz, H-8), 4.67 (dd, *J* = 6.1, 1.3 Hz, H-5), 4.07 (dd, *J* = 6.1, 3.1 Hz, H-6), 3.72 (m, H-9), 2.88 (dd, *J* = 13.2, 4.7 Hz, H-16), 2.73 (m, H-10), 2.72 (m, H-16), 2.55 (p, *J* = 2.8 Hz, H-14), 1.92 (td, *J* = 13.0, 5.0 Hz, H-15), 1.68 (m, H-15), 1.20 (s, H-10); ^13^C-NMR (150 MHz, DMSO-*d_6_*) δ 146.7 (C4), 139.2 (C3), 134.5 (C7), 130.7 (C12), 127.6 (C8), 124.7 (C11), 119.1 (C1), 117.1 (C2), 92.1 (C5), 66.4 (C6), 51.4 (C9), 43.6 (C13), 39.6 (C14), 37.9 (C16), 35.0 (C15), 29.2 (C10)

Dihydronormorphine (**2**) yield: 80%, m.p.: 265 °C (ethanol); ^1^H-NMR (600 MHz, DMSO-*d_6_*) δ 6.53 (d, *J* = 7.9 Hz, H-2), 6.42 (d, *J* = 8.0 Hz, H-1), 4.42 (d, *J* = 4.9 Hz, H-5), 3.81 (ddd, *J* = 8.6, 4.9, 3.5 Hz, H-6), 3.23 (dd, *J* = 6.2, 2.8 Hz, H-9), 2.79 (dd, *J* = 18.2, 6.3 Hz, H-10), 2.64 (m, H-16), 2.62 (m, H-10), 2.57 (m, *J*-16), 2.07 (ddd, *J* = 11.6, 6.7, 2.9 Hz, H-14), 1.69 (td, *J* = 12.5, 5.0 Hz, H-15), 1.48 (ddd, *J* = 12.5, 3.7, 1.5 Hz, H-15), 1.37 (m, H-8), 1.31 (m, H-7), 1.19 (m, H-7), 0.83 (tt, *J* = 12.2, 6.2 Hz, H-8); ^13^C-NMR (150 MHz, DMSO-*d_6_*) δ 146.4 (C4), 138.4 (C3), 130.3 (C12), 125.4 (C11), 118.4 (C1), 117.2 (C2), 90.5 (C5), 66.4 (C6), 52.3 (C9), 43.1 (C13), 38.6 (C14), 38.3 (C16), 37.7 (C15), 29.9 (C10), 26.3 (C7), 20.1 (C8)

14-*O*-Acetyloxycodone (2 mmol) was *N*-demethylated with α-chloro-ethyl chloroformate (10 mmol) for 16 h. The reaction was monitored by thin-layer chromatography to check the conversion, if it was necessary, another 5 mmol portion of α-chloro-ethyl chloroformate was added. The carbamate intermediate was decomposed in methanol to yield the hydrochloride salt of 14-*O*-acetylnoroxycodone. Acid hydrolysis in refluxing 10% HCl for 6 h resulted in the hydrochloride salt of noroxycodone. The free base of noroxycodone was liberated from the acid solution with 10% sodium hydroxide (pH = 10) and it was extracted with chloroform. The chloroform extract was dried under sodium sulfate and after evaporation of the solution, afforded the oily noroxycodone, which was rubbed with diethyl ether to produce crystalline material.

Noroxycodone (**6**) yield: 86%, m.p.: 163–166 °C; ^1^H-NMR (600 MHz, DMSO-*d_6_*) δ 6.70 (d, *J* = 8.2 Hz, H-2), 6.62 (d, *J* = 8.2 Hz, H-1), 4.70 (s, H-5), 3.78 (s, OCH_3_), 2.96 (m, H-10), 2.94 (m, H-9), 2.91 (m, H-7), 2.88 (m, H-10), 2.58 (dd, *J* = 13.3, 4.7 Hz, H-16), 2.34 (td, *J* = 12.6, 3.2 Hz, H-16), 2.30 (td, *J* = 12.0, 4.5 Hz, H-15), 2.08 (dt, *J* = 14.1, 3.2 Hz, H-7), 1.74 (ddd, *J* = 13.6, 5.1, 2.9 Hz, H-8), 1.40 (td, *J* = 14.1, 3.4 Hz, H-8), 1.14 (dd, *J* = 12.1, 3.0 Hz, H-15); ^13^C-NMR (150 MHz, DMSO-*d_6_*) δ 208.9 (C6), 144.9 (C4), 142.3 (C3), 130.3 (C12), 126.8 (C11), 119.5 (C1), 115.1 (C2), 90.5 (C5), 70.1 (C14), 57.3 (C9), 56.8 (OCH_3_), 51.0 (C13), 37.9 (C16), 36.3 (C7), 32.2 (C10), 32.0 (C8), 30.2 (C15)

*N*-Demethylation of 3,14-di-*O*-acetyloxymorphone yielded the hydrochloride salt of 3,14-di-*O*-acetylnoroxymorphone, which was hydrolyzed in 10% HCl solution. The noroxymorphone base was precipitated from the acid solution with 25% ammonia solution to yield noroxymorphone which is pure, for further reactions.

Noroxymorphone (**5**) yield: 81%, m.p.: >280 °C; ^1^H-NMR (600 MHz, DMSO-*d_6_*) δ 6.52 (d, *J* = 8.1 Hz, H-2), 6.49 (d, *J* = 8.1 Hz, H-1), 4.64 (s, H-5), 2.97 (m, H-9), 2.91 (m, H-10), 2.90 (m, H-7), 2.83 (m, H-10), 2.62 (dd, *J* = 13.1, 4.6 Hz, H-16), 2.37 (td, *J* = 12.8, 3.6 Hz, H-16), 2.29 (dd, *J* = 12.3, 4.8 Hz, H-15), 2.07 (dt, *J* = 14.2, 3.2 Hz, H-7), 1.74 (ddd, *J* = 13.6, 5.2, 2.9 Hz, H-8), 1.42 (td, *J* = 14.0, 3.5 Hz, H-8), 1.15 (dd, *J* = 12.1, 3.2 Hz, H-15); ^13^C-NMR (150 MHz, DMSO-*d_6_*) δ 209.1 (C6), 143.9 (C4), 139.8 (C3), 130.0 (C12), 124.4 (C11), 119.4 (C1), 117.6 (C2), 89.9 (C5), 70.1 (C14), 57.3 (C9), 50.9 (C13), 37.8 (C16), 36.3 (C7), 31.83 (C10), 31.77 (C8), 30.0 (C15)

#### 3.2.2. General Synthesis of *N*-Carboxymethyl-Nor-Compound Ethyl Esters

The appropriate normorphine derivative (2 mmol) was dissolved in 30 mL of acetonitrile. In the presence of sodium bicarbonate (10 mmol) ethyl bromoacetate (2.4 mmol) was added to the solution. The mixture was stirred and refluxed for 16 h. TLC monitoring showed that conversion was complete. The inorganic salts were filtered and the solvent was evaporated. Water (30 mL) was added to the residue and the pH was set around nine with cc. ammonia. Then it was extracted with chloroform (3 × 25 mL) and after unifying the organic phases, it was dried on sodium sulfate. After filtration the chloroform was evaporated. If it was necessary, column chromatography was used. (chloroform: methanol 9:1)

(**7**) *N*-carboxymethyl-normorphine ethyl ester (ethyl 2-((7aR)-7,9-dihydroxy-4,4a,7,7a-tetrahydro-1H-4,12-methanobenzofuro[3,2-e]isoquinolin-3(2H)-yl)acetate) yield: 86%, m.p.: 116 °C, HR-MS [M + H^+^]: calculated: 358.1649, measured: 358.1636; ^1^H-NMR (600 MHz, DMSO-*d_6_*) δ 6.40 (d, *J* = 8.0 Hz, H-2), 6.31 (d, *J* = 8.0 Hz, H-1), 5.49 (m, H-7), 5.19 (dt, *J* = 9.7, 2.8 Hz, H-8), 4.64 (dd, *J* = 6.1, 1.3 Hz, H-5), 4.09 (dd, *J* = 6.1, 3.1 Hz, H-6), 4.06 (q, *J* = 7.1 Hz, ester CH_2_), 3.39 (s, CH_2_ bridge), 3.35 (m, H-9), 3.23 (d, *J* = 16.5 Hz, CH_2_ bridge), 2.77 (d, *J* = 18.5 Hz, H-10) 2.58 (m, H-16), 2.55 (m, H-14), 2.31 (td, *J* = 12.2, 3.4 Hz, H-16), 2.26 (dd, *J* = 18.5, 6.4 Hz, H-10), 1.98 (m, H-15), 1.58 (dt, *J* = 11.0, 2.2 Hz, H-15), 1.16 (t, *J* = 7.1 Hz, ester CH_3_); ^13^C-NMR (150 MHz, DMSO-*d_6_*) δ 170.9 (ester C=O), 146.6 (C4), 138.9 (C3), 133.8 (C7), 131.3 (C12), 128.6 (C8), 125.7 (C11), 118.9 (C1), 116.7 (C2), 91.9 (C5), 66.7 (C6), 60.4 (ester CH_2_), 57.2 (C9), 56.5 (CH_2_ bridge), 44.9 (C16), 43.5 (C13), 40.66 (C14), 35.6 (C15), 22.4 (C10), 14.5 (ester CH_3_)

(**8**) *N*-carboxymethyl-dihydronormorphine ethyl ester (ethyl 2-((7aR)-7,9-dihydroxy-4,4a,5,6,7,7a-hexahydro-1H-4,12-methanobenzofuro[3,2-e]isoquinolin-3(2H)-yl)acetate) yield: 84%, m.p.: 86 °C, HR-MS [M + H^+^]: calculated: 360.1805, measured: 360.1789; ^1^H-NMR (600 MHz, DMSO-*d_6_*) δ 6.49 (d, *J* = 8.0 Hz, H-2), 6.38 (d, *J* = 8.0 Hz, H-1), 4.41 (d, *J* = 4.8 Hz, H-5), 4.04 (q, *J* = 7.1 Hz, ester CH_2_), 3.79 (dd, *J* = 9.1, 4.4 Hz, H-6), 3.31 (s, (CH_2_ bridge), 3.15 (d, *J* = 16.4 Hz, (CH_2_ bridge), 3.02 (dd, *J* = 6.0, 2.8 Hz, H-9), 2.71 (d, *J* = 18.3 Hz, H-10), 2.49 (m, H-16) 2.31 (dd, *J* = 18.4, 6.0 Hz, H-10), 2.18–2.09 (m, H-14, H-15), 1.78 (d, *J* = 5.1 Hz, H-15), 1.44–1.38 (m, H-15), 1.35 (dd, *J* = 13.4, 6.8 Hz, H-8), 1.28 (ddt, *J* = 12.8, 6.5, 3.9 Hz, H-7), 1.15 (t, *J* = 7.1 Hz, ester CH_3_), 1.11 (m, H-7), 0.83 (tt, *J* = 12.5, 6.4 Hz, H-8); ^13^C-NMR (150 MHz, DMSO-*d_6_*) δ 171.0 (ester C=O), 146.4 (C4), 138.4 (C3), 130.4 (C12), 125.4 (C11), 118.4 (C1), 117.1 (C2), 90.3 (C5), 66.5 (C6), 60.4 (ester CH_2_), 58.3 (C9), 56.8 (CH_2_ bridge), 44.9 (C16), 42.2 (C13), 38.2 (C14), 37.4 (C15), 26.0 (C7), 21.9 (C10), 19.9 (C8), 14.6 (ester CH_3_)

(**9**) *N*-carboxymethyl-norcodeine ethyl ester (ethyl 2-((7aR)-7-hydroxy-9-methoxy-4,4a,7,7a-tetrahydro-1H-4,12-methanobenzofuro[3,2-e]isoquinolin-3(2H)-yl)acetate) yield: 90%, m.p.: oil, HR-MS [M + H^+^]: calculated: 372.1805, measured: 372.1800; ^1^H-NMR (600 MHz, DMSO-*d_6_*) δ 6.58 (d, *J* = 8.2 Hz, H-2), 6.42 (d, *J* = 8.2 Hz, H-1), 5.49 (ddt, *J* = 9.8, 3.2, 1.5 Hz, H-7), 5.19 (dt, *J* = 9.8, 2.8 Hz, H-8), 4.66 (dd, *J* = 6.0, 1.3 Hz, H-5), 4.10 (tq, *J* = 5.7, 2.7 Hz, H-6), 4.05 (q, *J* = 7.1 Hz, ester CH_2_), 3.70 (d, *J* = 2.2 Hz, CH_2_ bridge), 3.69 (s, OCH_3_), 3.23 (d, *J* = 16.6 Hz, CH_2_ bridge), 2.81 (d, *J* = 18.6 Hz, H-10), 2.57 (dp, *J* = 8.8, 3.3, 2.6 Hz, H-16), 2.56 (m, H-14), 2.34–2.25 (m, H-10, H-16), 1.98 (td, *J* = 12.6, 5.0 Hz, H-15), 1.62–1.53 (m, H-15), 1.16 (t, *J* = 7.1 Hz, ester CH_3_); ^13^C-NMR (150 MHz, DMSO-*d_6_*) δ 170.6 (ester C=O), 147.2 (C4), 141,7 (C3), 133.9 (C7), 131.6 (C12), 128.7 (C8), 127.5 (C11), 118.9 (C1), 113.7 (C2), 92.5 (C5), 67.0 (C6), 60.5 (ester CH_2_), 57.3 (C9), 56.6 (CH_2_ bridge), 56.5 (OCH_3_), 44.9 (C16), 43.5 (C13), 40.6 (C14), 35.6 (C15), 22.6 (C10), 14.6 (ester CH_3_)

(**10**) *N*-carboxymethyl-dihydronorcodeine ethyl ester (ethyl 2-((7aR)-7-hydroxy-9-methoxy-4,4a,5,6,7,7a-hexahydro-1H-4,12-methanobenzofuro[3,2-e]isoquinolin-3(2H)-yl)acetate) yield: 88%, m.p.: oil, HR-MS [M + H^+^]: calculated: 374.1961, measured: 374.1959; ^1^H-NMR (600 MHz, DMSO-*d_6_*) δ 6.66 (d, *J* = 8.2 Hz, H-2), 6.50 (d, *J* = 8.2 Hz, H-1), 4.43 (dd, *J* = 7.8, 4.9 Hz, H-5), 4.04 (q, *J* = 7.1 Hz, ester CH_2_), 3.79 (tt, *J* = 8.7, 4.1 Hz, H-6), 3.72 (s, OCH_3_), 3.32 (s, CH_2_ bridge), 3.16 (d, *J* = 16.4 Hz, CH_2_ bridge), 3.04 (dd, *J* = 5.9, 2.7 Hz, H-9), 2.75 (d, *J* = 18.4 Hz, H-10), 2.51–2.48 (m, H-16), 2.34 (dd, *J* = 18.4, 6.0 Hz, H-10), 2.17–2.09 (m, H-16, H-14), 1.79 (td, *J* = 12.4, 5.0 Hz, H-15), 1.40 (ddd, *J* = 12.5, 3.6, 1.7 Hz, H-15), 1.34 (dd, *J* = 13.3, 6.7 Hz, H-8), 1.29 (ddd, *J* = 13.4, 7.2, 4.2 Hz, H-7), 1.15 (t, *J* = 7.1 Hz, ester CH_3_), 1.13–1.08 (m, H-7), 0.84 (tt, *J* = 11.9, 6.1 Hz, H-8); ^13^C-NMR (150 MHz, DMSO-*d_6_*) δ 170.9 (ester C=O), 147.4 (C4), 141.3 (C3), 130.7 (C12), 127.3 (C11), 118.4 (C1), 114.4 (C2), 90.7 (C5), 66.3 (C6), 60.3 (ester CH_2_), 58.2 (C9), 56.8 (CH_2_ bridge), 56.7 (OCH_3_), 44.9 (C16), 42.4 (C13), 38.4 (C14), 37.3 (C15), 26.1 (C7), 22.0 (C10), 19.8 (C8), 14.5 (ester CH_3_)

(**11**) *N*-carboxymethyl-noroxymorphone ethyl ester (ethyl 2-((7aR)-4a,9-dihydroxy-7-oxo-4,4a,5,6,7,7a-hexahydro-1H-4,12-methanobenzofuro[3,2-e]isoquinolin-3(2H)-yl)acetate) yield: 82%, m.p.: 160 °C, HR-MS [M + H^+^]: calculated: 374.1598, measured: 374.1588; ^1^H-NMR (600 MHz, DMSO-*d_6_*) δ 6.53 (d, *J* = 8.1 Hz, H-2), 6.50 (d, *J* = 8.1 Hz, H-1), 4.74 (s, H-5), 4.12–4.06 (m, ester CH_2_), 3.43 (d, *J* = 17.1 Hz, CH_2_ bridge), 3.31 (d, *J* = 17.2 Hz, CH_2_ bridge), 2.97 (d, *J* = 18.5 Hz, H-10), 2.90 (d, *J* = 5.9 Hz, H-9), 2.89–2.82 (m, H-7), 2.57 (d, *J* = 5.9 Hz, H-10), 2.49 (m, H-16), 2.38–2.29 (m, H-15), 2.18 (td, *J* = 12.0, 3.6 Hz, H-16), 2.06 (dt, *J* = 14.2, 3.2 Hz, H-7), 1.71 (ddd, *J* = 13.4, 5.0, 3.0 Hz, H-8), 1.40 (td, *J* = 14.1, 3.4 Hz, H-8), 1.28–1.22 (m, H-15), 1.18 (t, *J* = 7.1 Hz, ester CH_3_); ^13^C-NMR (150 MHz, DMSO-*d_6_*) δ 209.0 (C6), 171.3 (ester C=O), 143.8 (C4), 139.8 (C3), 129.6 (C12), 123.6 (C11), 119.5 (C1), 117.6 (C2), 89.7 (C5), 70.4 (C14), 62.8 (C9), 60.7 (ester CH_2_), 55.6 (CH_2_ bridge), 50.3 (C13), 43.9 (C16), 36.2 (C7), 31.5 (C8), 30.6 (C15), 24.3 (C10), 14.5 (ester CH_3_)

(**12**) *N*-carboxymethyl-noroxycodone ethyl ester (ethyl 2-((7aR)-4a-hydroxy-9-methoxy-7-oxo-4,4a,5,6,7,7a-hexahydro-1H-4,12-methanobenzofuro[3,2-e]isoquinolin-3(2H)-yl)acetate) yield: 87%, m.p.: 144 °C, HR-MS [M + H^+^]: calculated: 388.1755, measured: 388.1752; ^1^H-NMR (600 MHz, DMSO-*d_6_*) δ 6.72 (d, *J* = 8.2 Hz, H-2), 6.63 (d, *J* = 8.2 Hz, H-1), 4.81 (s, H-5), 4.15–4.03 (m, ester CH_2_), 3.75 (s, OCH_3_), 3.44 (d, *J* = 17.1 Hz, CH_2_ bridge), 3.32 (d, CH_2_ bridge), 3.01 (d, *J* = 18.6 Hz, H-10), 2.92 (d, *J* = 5.7 Hz, H-9), 2.87 (td, *J* = 14.4, 5.0 Hz, H-7), 2.59 (dd, *J* = 18.7, 5.8 Hz, H-10), 2.52–2.48 (m, H-16), 2.34 (td, *J* = 12.5, 5.3 Hz, H-15), 2.17 (td, *J* = 12.0, 3.6 Hz, H-16), 2.06 (dt, *J* = 14.1, 3.2 Hz, H-7), 1.72 (ddd, *J* = 13.5, 5.0, 3.0 Hz, H-8), 1.43–1.34 (m, H-8), 1.30–1.23 (m, H-15), 1.18 (t, *J* = 7.1 Hz, ester CH_3_); ^13^C-NMR (150 MHz, DMSO-*d_6_*) δ 208.8 (C6), 171.3 (ester C=O), 144.8 (C4), 142.4 (C3), 129,9 (C12), 125.8 (C11), 119.7 (C1), 115.2 (C2), 90.2 (C5), 70.3 (C14), 62.8 (C9), 60.7 (ester CH_2_), 56.8 (OCH_3_), 55.7 (CH_2_ bridge), 50.2 (C13), 43.8 (C16), 36.3 (C7), 31.6 (C8), 30.5 (C15), 24.3 (C16), 14.5 (ester CH_3_)

#### 3.2.3. General Synthesis of *N*-Carboxymethyl-Nor-Compounds

The *N*-carboxymethyl-nor-compound ethyl ester (0.5 mmol) was added to the mixture of ethanol (1 g) and water (2.5 mL). To this solution, sodium hydroxide (1 M, 0.5 mmol) was added and the reaction mixture was heated for 1 h at 60 °C. The hydrolysis reaction was monitored by TLC and upon complete disappearance of the starting material, the pH was adjusted to 3–4 with 10% HCl solution. Then the solvent was evaporated to obtain the hydrochloride salt of the desired compound.

(**19**) *N*-carboxymethyl-normorphine HCl (2-((7aR)-7,9-dihydroxy-4,4a,7,7a-tetrahydro-1H-4,12-methanobenzofuro[3,2-e]isoquinolin-3(2H)-yl)acetic acid hydrochloride) yield: 92%, m.p.: > 270 °C decomp., HR-MS [M + H^+^]: calculated: 330.1336, measured: 330.1332; ^1^H-NMR (600 MHz, D_2_O) δ 6.61 (d, *J* = 8.2 Hz, H-2), 6.53 (d, *J* = 8.2 Hz, H-1), 5.60 (m, H-7), 5.24 (d, *J* = 10.0 Hz, H-8), 4.92 (d, *J* = 6.5 Hz, H-5), 4.27 (m, H-9), 4.25 (m, H-6), 4.00 (m, CH_2_ bridge) 3.48 (q, *J* = 7.1 Hz, H-16), 3.11 (d, *J* = 20.1 Hz, H-10), 2.99 (d, *J* = 18.1 Hz, H-14), 2.80 (dd, *J* = 20.0, 6.8 Hz, H-10), 2.39–2.19 (m, H-15), 2.02 (d, *J* = 14.6 Hz, H-13); ^13^C-NMR (150 MHz, D_2_O) δ 169.2 (ester C=O), 145.5 (C4), 137.8 (C3), 132.9 (C7), 129.0 (C12), 125.4 (C8), 123.0 (C11), 120.0 (C1), 117.6 (C2), 89.9 (C5), 65.5 (C6), 60.2 (C9), 54.9 (CH_2_ bridge), 47.1 (C16), 41.8 (C13), 38.1 (C14), 32.2 (C15), 21.9 (C16)

(**20**) *N*-carboxymethyl-dihydronormorphine HCl (2-((7aR)-7,9-dihydroxy-4,4a,5,6,7,7a-hexahydro-1H-4,12-methanobenzofuro[3,2-e]isoquinolin-3(2H)-yl)acetic acid hydrochloride) yield: 93%, m.p.: > 315 °C decomp., HR-MS [M + H^+^]: calculated: 332.1492, measured: 332.1482; ^1^H-NMR (600 MHz, D_2_O) δ 6.69 (d, *J* = 7.9 Hz, H-2), 6.62 (d, *J* = 8.2 Hz, H-1), 4.64 (m, H-5), 4.07–3.83 (m, H-6, H-9, CH_2_ bridge), 3.34 (dd, *J* = 13.4, 4.4 Hz, H-16), 3.06 (d, *J* = 20.2 Hz, H-10), 2.91 (dd, *J* = 19.9, 6.0 Hz, H-10), 2.82 (ddd, *J* = 17.3, 13.9, 7.6 Hz, H-16), 2.47 (t, *J* = 19.4 Hz, H-14), 2.08 (td, *J* = 13.9, 5.0 Hz, H-15), 1.78 (dd, *J* = 14.7, 3.7 Hz, H-15), 1.44 (q, *J* = 6.2 Hz, H-7, H-8), 0.99–0.83 (m, H-8); ^13^C-NMR (150 MHz, D_2_O) δ 168.3 (carboxylic C=O), 145.2 (C4), 137.1 (C3), 128.4 (C12), 122.6 (C11), 119.8 (C1), 117.8 (C2), 89.0 (C5), 66.1 (C6), 61.5 (C9), 55.0 (CH_2_ bridge), 47.5 (C16), 40.2 (C13), 38.3 (C14), 34.1 (C15), 26.0 (C7), 21.4 (C10), 17.7 (C8)

(**21**) *N*-carboxymethyl-norcodeine HCl (2-((7aR)-7-hydroxy-9-methoxy-4,4a,7,7a-tetrahydro-1H-4,12-methanobenzofuro[3,2-e]isoquinolin-3(2H)-yl)acetic acid hydrochloride) yield: 95%, m.p.: > 260 °C decomp., HR-MS [M + H^+^]: calculated: 344.1492, measured: 344.1481; ^1^H-NMR (600 MHz, D_2_O) δ 6.79 (d, *J* = 8.3 Hz, H-2), 6.65 (d, *J* = 8.3 Hz, H1), 5.68–5.54 (m, H-7), 5.33–5.18 (m, H-8), 5.03–4.87 (m, H-5), 4.39–3.94 (m, H-9, H-6, CH_2_ bridge), 3.72 (s, OCH_3_), 3.55–3.40 (m, H-16), 3.16 (d, *J* = 20.1 Hz, H-10), 3.10–2.73 (m, H-14, H-10), 2.31 (s, H-15), 2.05 (d, *J* = 14.9 Hz, H-15); ^13^C-NMR (150 MHz, D_2_O) δ 168.8 (carboxylic C=O), 146.4 (C4), 141.9 (C3), 133.1 (C7), 128.9 (C12), 125.5 (C8), 124.0 (C11), 120.4 (C1), 114.7 (C2), 90.6 (C5), 65.9 (C6), 60.5 (C9), 56.4 (OCH_3_) 54.8 (CH_2_ bridge), 46.8 (C16), 41.7 (C13), 38.4 (C14), 32.4 (C15), 21.9

(**22**) *N*-carboxymethyl-dihydronorcodeine HCl (2-((7aR)-7-hydroxy-9-methoxy-4,4a,5,6,7,7a-hexahydro-1H-4,12-methanobenzofuro[3,2-e]isoquinolin-3(2H)-yl)acetic acid hydrochloride) yield: 90%, m.p.: > 275 °C decomp., HR-MS [M + H^+^]: calculated: 346.1649, measured: 346.1644; ^1^H-NMR (600 MHz, D_2_O) δ 6.85 (d, *J* = 8.2 Hz, H-2), 6.72 (d, *J* = 8.2 Hz, H-1), 4.69 (d, *J* = 5.4 Hz, H-5), 4.16–3.79 (m, H-6, H-9, CH_2_ bridge), 3.80–3.70 (s, OCH_3_), 3.38–3.29 (m, H-16), 3.09 (d, *J* = 20.2 Hz, H-10), 2.99–2.87 (m, H-10), 2.80 (t, *J* = 12.6 Hz, H-16), 2.46 (d, *J* = 11.6 Hz, H-14), 2.08 (dd, *J* = 14.7, 10.0 Hz, H-15), 1.78 (d, *J* = 14.0 Hz, H-15), 1.45 (d, *J* = 8.7 Hz, H-7, H-8), 0.98–0.87 (m, H-8); ^13^C-NMR (150 MHz, D_2_O) δ 168.5 (carboxylic C=O), 145.8 (C4), 141.3 (C3), 127.8 (C12), 123.4 (C11), 119.9 (C1), 115.0 (C2), 89.2 (C5), 66.0 (C6), 61.5 (C9), 56.7 (OCH_3_), 55.1 (CH_2_ bridge), 47.4 (C16), 40.1 (C13), 38.2 (C14), 34.1 (C15), 26.0 (C7), 21.4 (C10), 17.6 (C8)

(**23**) *N*-carboxymethyl-noroxymorphone HCl (2-((7aR)-4a,9-dihydroxy-7-oxo-4,4a,5,6,7,7a-hexahydro-1H-4,12-methanobenzofuro[3,2-e]isoquinolin-3(2H)-yl)acetic acid hydrochloride) yield: 96%, m.p.: > 290 °C decomp., HR-MS [M + H^+^]: calculated: 346.1285, measured: 346.1283; ^1^H-NMR (600 MHz, D_2_O) δ 6.70 (d, *J* = 8.2 Hz, H-2), 6.68 (d, *J* = 8.3 Hz, H-1), 4.92 (s, H-5), 3.93 (d, *J* = 16.7 Hz, CH_2_ bridge), 3.88 (d, *J* = 6.2 Hz, H-9), 3.69 (d, *J* = 16.7 Hz, CH_2_ bridge), 3.29 (d, *J* = 20.0 Hz, H-10), 3.18 (dd, *J* = 13.1, 4.9 Hz, H-16), 3.06 (dd, *J* = 20.1, 6.4 Hz, H-10), 2.89 (td, *J* = 14.8, 5.1 Hz, H-7), 2.81 (td, *J* = 13.0, 4.1 Hz, H-16), 2.65 (td, *J* = 13.5, 5.0 Hz, H-15), 2.20 (dt, *J* = 14.9, 3.2 Hz, H-7), 1.96 (ddd, *J* = 14.6, 5.2, 2.9 Hz, H-8), 1.65 (dd, *J* = 12.9, 3.7 Hz, H-15), 1.60 (dd, *J* = 14.6, 3.6 Hz, H-8); ^13^C-NMR (150 MHz, D_2_O) δ 209.3 (C6), 168.8 (carboxylic C=O), 143.0 (C4), 138.5 (C3), 127.2 (C12), 121.7 (C11), 121.1 (C1), 118.7 (C2), 89.2 (C5), 70.7 (C14), 64.9 (C9), 54.7 (CH_2_ bridge), 48.6 (C13), 46.7 (C16), 34.6 (C7), 30.5 (C8), 27.5 (C15), 23.9 (C10)

(**24**) *N*-carboxymethyl-noroxycodone HCl (2-((7aR)-4a-hydroxy-9-methoxy-7-oxo-4,4a,5,6,7,7a-hexahydro-1H-4,12-methanobenzofuro[3,2-e]isoquinolin-3(2H)-yl)acetic acid hydrochloride) yield: 94%, m.p.: > 210 °C decomp., HR-MS [M + H^+^]: calculated: 360.1442, measured: 360.1435; ^1^H-NMR (600 MHz, D_2_O) δ 6.86 (d, *J* = 8.4 Hz, H-2), 6.78 (d, *J* = 8.4 Hz, H-1), 4.95 (s, H-5), 3.94–3.79 (m, H-9, CH_2_ bridge), 3.74 (s, OCH_3_), 3.63 (d, *J* = 16.4 Hz, CH_2_ bridge), 3.31 (d, *J* = 20.0 Hz, H-10), 3.17 (ddt, *J* = 13.1, 5.0, 1.4 Hz, H-16), 3.12–3.05 (m, H-10), 2.90 (td, *J* = 14.8, 5.1 Hz, H-7), 2.80 (td, *J* = 13.0, 4.1 Hz, H-16), 2.65 (td, *J* = 13.5, 5.0 Hz, H-15), 2.21 (dt, *J* = 14.9, 3.2 Hz, H-7), 1.96 (ddd, *J* = 14.5, 5.1, 2.9 Hz, H-8), 1.65 (dd, *J* = 14.2, 3.8 Hz, H-15), 1.60 (dd, *J* = 14.6, 3.5 Hz, H-8); ^13^C-NMR (150 MHz, D_2_O) δ 210.3 (C6), 169.2 (carboxylic C=O), 143.9 (C4), 142.4 (C3), 127.1 (C12), 122.5 (C11), 121.1 (C1), 115.6 (C2), 89.4 (C5), 70.4 (C14), 64.9 (C9), 56.7 (OCH_3_), 55.2 (CH_2_ bridge), 48.7 (C13), 46.6 (C16), 34.5 (C7), 30.5 (C8), 27.6 (C15), 23.9 (C10)

#### 3.2.4. General Synthesis of *N*-Carboxyethyl-Nor-Compound Ethyl Esters

The normorphine derivative (2 mmol) was dissolved in 30 mL of ethanol. In the presence of triethylamine (1 mL) ethyl acrylate (2.4 mmol) was added to the solution. The mixture was stirred and refluxed for 3 h. After the TLC showed no more starting compound, the solvent was evaporated. If it was necessary, column chromatography was used. (chloroform:methanol 9:1)

(**13**) *N*-carboxyethyl-normorphine ethyl ester (ethyl 3-((7aR)-7,9-dihydroxy-4,4a,7,7a-tetrahydro-1H-4,12-methanobenzofuro[3,2-e]isoquinolin-3(2H)-yl)propanoate) yield: 98%, m.p.: 158 °C, HR-MS [M + H^+^]: calculated: 372.1805, measured: 372.1798; ^1^H-NMR (600 MHz, DMSO-*d_6_*) δ 6.44 (d, *J* = 8.0 Hz, H-2), 6.34 (d, *J* = 8.1 Hz, H-1), 5.52 (dp, *J* = 9.7, 1.5 Hz, H-7), 5.22 (dt, *J* = 9.8, 2.8 Hz, H-8), 4.66 (dd, *J* = 6.1, 1.3 Hz, H-5), 4.15–4.00 (m, H-6, ester CH_2_), 3.35 (m, H-9), 2.78 (dt, *J* = 14.3, 5.9 Hz, H-10, ethylene bridge CH_2_), 2.64 (dt, *J* = 12.9, 6.6 Hz, ethylene bridge CH_2_), 2.60–2.53 (m, H-16), 2.48–2.39 (m, H-14, ethylene bridge CH_2_), 2.33–2.21 (m, H-16, H-10), 1.92 (td, *J* = 12.5, 5.0 Hz, H-15), 1.61 (ddd, *J* = 12.5, 3.4, 1.7 Hz, H-15), 1.19 (t, *J* = 7.1 Hz, ester CH_3_); ^13^C-NMR (150 MHz, DMSO-*d_6_*) δ 172.3 (ester C=O), 146.8 (C4), 138.7 (C3), 133.9 (C7), 131.2 (C12), 128.8 (C8), 125.7 (C11), 118.9 (C1), 116.8 (C2), 92.0 (C5), 66.8 (C6), 60.3 (ester CH_2_), 57.1 (C9), 50.8 (ethylene bridge CH_2_), 44.6 (C16), 43.8 (C13), 41.6 (C14), 36.0 (C15), 33.7 (ethylene bridge CH_2_), 22.2 (C10), 14.7 (ester CH_3_)

(**14**) *N*-carboxyethyl-dihydronormorphine ethyl ester (ethyl 3-((7aR)-7,9-dihydroxy-4,4a,5,6,7,7a-hexahydro-1H-4,12-methanobenzofuro[3,2-e]isoquinolin-3(2H)-yl)propanoate) yield: 96%, m.p.: 120 °C, HR-MS [M + H^+^]: calculated: 374.1961, measured: 374.1960; ^1^H-NMR (600 MHz, DMSO-*d_6_*) δ 6.53 (d, *J* = 8.0 Hz, H-2), 6.41 (d, *J* = 8.0 Hz, H-1), 4.44 (d, *J* = 4.8 Hz, H-5), 4.06 (q, *J* = 7.1 Hz, ester CH_2_), 3.80 (dq, *J* = 8.7, 4.2 Hz, H-6), 3.16–2.99 (m, H-9), 2.77 (d, *J* = 18.5 Hz, H-10), 2.46 (d, *J* = 6.9 Hz, ethylene bridge CH_2_), 2.12 (d, *J* = 39.2 Hz, H-16), 1.82–1.72 (m, H-15), 1.46 (d, *J* = 12.4 Hz, H-15), 1.38 (dq, *J* = 13.5, 6.9 Hz, H-8), 1.30 (dtd, *J* = 13.2, 6.5, 3.4 Hz, H-7), 1.18 (td, *J* = 7.2, 4.2 Hz, H-7, ester CH_3_), 0.87 (tt, *J* = 12.6, 6.4 Hz, H-8); ^13^C-NMR (150 MHz, DMSO-*d_6_*) δ 172.2 (ester C=O), 146.5 (C4), 138.3 (C3), 130.1 (C12), 125.2 (C11), 117.8 (C1), 116.6 (C2), 89.6 (C5), 65.8 (C6), 59.6 (ester CH_2_), 57.4 (C9), 49.9 (ethylene bridge CH_2_), 44.0 (C16), 42.7 (C13), 37.6 (C14), 36.7 (C15), 32.6 (ethylene bridge CH_2_), 25.4 (C7), 20.9 (C10), 19.3 (C8), 14.2 (ester CH_3_)

(**15**) *N*-carboxyethyl-norcodeine ethyl ester (ethyl 3-((7aR)-7-hydroxy-9-methoxy-4,4a,7,7a-tetrahydro-1H-4,12-methanobenzofuro[3,2-e]isoquinolin-3(2H)-yl)propanoate) yield: 97%, m.p.: oil, HR-MS [M + H^+^]: calculated: 386.1962, measured: 386.1951; ^1^H-NMR (600 MHz, DMSO-*d_6_*) δ 6.61 (d, *J* = 8.2 Hz, H-2), 6.45 (d, *J* = 8.2 Hz, H-1), 5.53 (dp, *J* = 9.8, 1.5 Hz, H-7), 5.23 (dt, *J* = 9.8, 2.8 Hz, H-8), 4.67 (dd, *J* = 5.9, 1.3 Hz, H-5), 4.11 (dt, *J* = 5.7, 2.9 Hz, H-6), 4.07 (q, *J* = 7.1 Hz, ester CH_2_), 3.71 (s, OCH_3_), 3.36 (s, H-9), 2.86–2.74 (m, H-10, ethylene bridge), 2.64 (dt, *J* = 12.9, 6.7 Hz, ethylene bridge), 2.61–2.53 (m, H-16), 2.49–2.42 (m, H-14, ethylene bridge), 2.31 (dd, *J* = 18.6, 6.3 Hz, H-10), 2.24 (td, *J* = 12.2, 3.4 Hz, H-16), 1.92 (td, *J* = 12.5, 5.0 Hz, H-15), 1.61 (ddd, *J* = 12.6, 3.4, 1.7 Hz, H-15), 1.18 (t, *J* = 7.1 Hz, ester CH_3_); ^13^C-NMR (150 MHz, DMSO-*d_6_*) δ 171.7 (ester C=O), 145.0 (C4), 141.2 (C3), 133.1 (C7), 130.7 (C12), 128.0 (C8), 127.0 (C11), 118.1 (C1), 113.0 (C2), 91.7 (C5), 66.2 (C6), 59.5 (ester CH_2_), 56.2 (C9), 55.8 (OCH_3_), 49.9 (ethylene bridge CH_2_), 43.7 (C16), 43.4 (C13), 40.2 (C14), 35.2 (C15), 32.9 (ethylene bridge CH_2_), 21.5 (C10), 14.0 (ester CH_3_)

(**16**) *N*-carboxyethyl-dihydronorcodeine ethyl ester (ethyl 3-((7aR)-7-hydroxy-9-methoxy-4,4a,5,6,7,7a-hexahydro-1H-4,12-methanobenzofuro[3,2-e]isoquinolin-3(2H)-yl)propanoate) yield: 93%, m.p.: oil, HR-MS [M + H^+^]: calculated: 388.2118, measured: 388.2111; ^1^H-NMR (600 MHz, DMSO-*d_6_*) δ 6.69 (d, *J* = 8.2 Hz, H-2), 6.53 (d, *J* = 8.1 Hz, H-1), 4.45 (dd, *J* = 6.7, 4.9 Hz, H-5), 4.06 (qd, *J* = 7.1, 1.2 Hz, ester CH2), 3.84–3.77 (m, H-6), 3.76 (s, OCH_3_), 3.04 (dd, *J* = 5.9, 2.8 Hz, H-9), 2.81–2.70 (m, H-10, ethylene bridge CH_2_), 2.59 (dq, *J* = 12.8, 6.6, 5.9 Hz, ethylene bridge CH_2_), 2.46–2.38 (m, ethylene bridge CH_2_), 2.36 (dd, *J* = 18.4, 6.0 Hz, H-10), 2.12–2.02 (m, H-14, H-16), 1.74 (ddd, *J* = 14.3, 11.2, 4.9 Hz, H-15), 1.44 (ddd, *J* = 12.4, 3.6, 1.7 Hz, H-15), 1.37 (dt, *J* = 13.4, 6.8 Hz, H-8), 1.31 (dq, *J* = 10.0, 3.5 Hz, H-7), 1.18 (t, *J* = 7.1 Hz, ester CH_3_), 1.16–1.11 (m, H-8), 0.94–0.82 (m, H-7); ^13^C-NMR (150 MHz, DMSO-*d_6_*) δ 172.4 (ester C=O), 147.4 (C4), 140.9 (C3), 130.7 (C12), 127.2 (C11), 118.4 (C1), 114.4 (C2), 90.7 (C5), 66.3 (C6), 60.1 (ester CH_2_), 57.9 (C9), 56.7 (OCH_3_), 50.6 (ethylene bridge CH_2_), 44.5 (C16), 42.6 (C13), 38.6 (C14), 37.5 (C15), 33.6 (ethylene bridge CH_2_), 26.1 (C7), 21.6 (C10), 19.8 (C8), 14.6 (ester CH_3_)

(**17**) *N*-carboxyethyl-noroxymorphone ethyl ester (ethyl 3-((7aR)-4a,9-dihydroxy-7-oxo-4,4a,5,6,7,7a-hexahydro-1H-4,12-methanobenzofuro[3,2-e]isoquinolin-3(2H)-yl)propanoate) yield: 99%, m.p.: 137 °C, HR-MS [M + H^+^]: calculated: 388.1754, measured: 388.1740; ^1^H-NMR (600 MHz, DMSO-*d_6_*) δ 6.56 (d, *J* = 8.1 Hz, H-2), 6.52 (d, *J* = 8.1 Hz, H-1), 4.75 (s, H-5), 4.08 (qd, *J* = 7.1, 2.8 Hz, ester CH2), 2.97 (d, *J* = 18.5 Hz, H-10), 2.91 (d, *J* = 5.9 Hz, H-9), 2.90–2.84 (m, H-7), 2.75 (dt, *J* = 12.7, 7.2 Hz, ethylene bridge CH_2_), 2.67 (dt, *J* = 12.6, 6.2 Hz, ethylene bridge CH_2_), 2.60–2.53 (m, H-10), 2.53–2.51 (m, H-16), 2.27 (td, *J* = 12.6, 5.1 Hz, H-15), 2.08 (dt, *J* = 14.1, 3.2 Hz, H-7), 2.02 (td, *J* = 12.1, 3.7 Hz, H-16), 1.74 (ddd, *J* = 13.3, 5.0, 2.9 Hz, H-8), 1.43 (td, *J* = 14.0, 3.4 Hz, H-8), 1.32–1.25 (m, H-15), 1.21 (t, *J* = 7.1 Hz, ester CH_3_); ^13^C-NMR (150 MHz, DMSO-*d_6_*) δ 208.7 (C6), 172.0 (ester C=O), 143.4 (C4), 139.4 (C3), 129.4 (C12), 123.3 (C11), 119.0 (C1), 117.2 (C2), 89.3 (C5), 69.8 (C14), 62.6 (C9), 59.9 (ester CH_2_), 50.1 (C13), 49.7 (ethylene bridge CH_2_), 42.7 (C16), 35.8 (C7), 32.9 (ethylene bridge CH_2_), 31.1 (C8), 30.2 (C15), 23.1 (C10), 14.2 (ester CH_3_)

(**18**) *N*-carboxyethyl-noroxycodone ethyl ester (ethyl 3-((7aR)-4a-hydroxy-9-methoxy-7-oxo-4,4a,5,6,7,7a-hexahydro-1H-4,12-methanobenzofuro[3,2-e]isoquinolin-3(2H)-yl)propanoate) yield: 98%, m.p.: 119 °C, HR-MS [M + H^+^]: calculated: 402.1911, measured: 402.1905; ^1^H-NMR (600 MHz, DMSO-*d_6_*) δ 6.74 (d, *J* = 8.3 Hz, H-2), 6.66 (d, *J* = 8.2 Hz, H-1), 4.83 (d, *J* = 7.8 Hz, H-5), 4.09 (qd, *J* = 7.1, 2.8 Hz, ester CH_2_), 3.78 (s, OCH_3_), 3.02 (d, *J* = 18.6 Hz, H-10), 2.94 (d, *J* = 5.7 Hz, H-9), 2.89 (d, *J* = 5.0 Hz, H-7), 2.81–2.72 (m, ethylene bridge CH_2_), 2.68 (dt, *J* = 12.6, 6.2 Hz, ethylene bridge CH_2_), 2.59 (dd, *J* = 18.7, 5.8 Hz, H-10), 2.57–2.52 (m, ethylene bridge CH_2_), 2.28 (td, *J* = 12.6, 5.2 Hz, H-15), 2.08 (dt, *J* = 14.1, 3.2 Hz, H-7), 2.00 (td, *J* = 12.2, 3.7 Hz, H-16), 1.75 (ddd, *J* = 13.4, 5.0, 3.0 Hz, H-8), 1.46–1.37 (m, H-8), 1.33–1.26 (m, H-15), 1.21 (t, *J* = 7.1 Hz, ester CH_3_); ^13^C-NMR (150 MHz, DMSO-*d_6_*) δ 208.3 (C6), 172.1 (ester C=O), 144.2 (C4), 141.9 (C3), 129.5 (C12), 125.5 (C11), 118.9 (C1), 114.4 (C2), 89.4 (C5), 69.8 (C14), 62.5 (C9), 59.4 (ester CH_2_), 55.9 (OCH_3_), 50.2 (C13), 49.2 (ethylene bridge CH_2_), 42.7 (C16), 35.5 (C7), 32.4 (ethylene bridge CH_2_), 30.8 (C8), 29.7 (C15), 22.8 (C10), 13.9 (ester CH_3_)

#### 3.2.5. General Synthesis of *N*-Carboxyethyl-Nor-Compounds

The *N*-carboxyethyl-nor-compound ethyl ester (0.5 mmol) was added to the mixture of ethanol (1 g) and water (2.5 mL). To this solution, sodium hydroxide (1 M, 0.5 mmol) was added and the reaction mixture was heated for 1 h at 60 °C. The hydrolysis reaction was monitored by TLC and upon complete disappearance of the starting material the pH was adjusted to 3–4 with 10% HCl solution. Then, the solvent was evaporated to obtain the hydrochloride salt of the desired compound.

(**25**) *N*-carboxyethyl-normorphine HCl (3-((7aR)-7,9-dihydroxy-4,4a,7,7a-tetrahydro-1H-4,12-methanobenzofuro[3,2-e]isoquinolin-3(2H)-yl)propanoic acid hydrochloride) yield: 96%, m.p.: > 290 °C decomp., HR-MS [M + H^+^]: calculated: 344.1492, measured: 344.1483; ^1^H-NMR (600 MHz, D_2_O) δ 6.64 (d, *J* = 8.2 Hz, H-2), 6.56 (d, *J* = 8.2 Hz, H-1), 5.62 (d, *J* = 9.7 Hz, H-7), 5.27 (d, *J* = 10.1 Hz, H-8), 4.94 (dd, *J* = 6.4, 1.3 Hz, H-5), 4.29–4.18 (m, H-6, H-9), 3.48–3.33 (m, H-16, ethylene bridge CH_2_), 3.13 (d, *J* = 20.1 Hz, H-10), 2.96 (t, *J* = 13.2 Hz, H-16), 2.89–2.78 (m, H-14, H-10), 2.71 (td, *J* = 7.0, 2.7 Hz, ethylene bridge CH_2_), 2.22 (t, *J* = 13.8 Hz, H-15), 2.04 (d, *J* = 14.3 Hz, H-15); ^13^C-NMR (150 MHz, D_2_O) δ 175.3 (carboxylic C=O), 145.1 (C4), 137.8 (C3), 129.3 (C12), 133.1 (C7), 125.5 (C8), 123.2 (C11), 120.3 (C1), 117.6 (C2), 90.3 (C5), 65.6 (C6), 58.8 (C9), 50.7 (ethylene bridge CH_2_), 46.0 (C16), 41.8 (C13), 38.1 (C14), 32.5 (C15), 29.6 (ethylene bridge CH_2_), 21.2 (C10)

(**26**) *N*-carboxyethyl-dihydronormorphine HCl (3-((7aR)-7,9-dihydroxy-4,4a,5,6,7,7a-hexahydro-1H-4,12-methanobenzofuro[3,2-e]isoquinolin-3(2H)-yl)propanoic acid hydrochloride) yield: 96%, m.p.: > 295 °C decomp., HR-MS [M + H^+^]: calculated: 346.1649, measured: 346.1647; ^1^H-NMR (600 MHz, D_2_O) δ 6.70 (dd, *J* = 8.2, 0.9 Hz, H-2), 6.63 (d, *J* = 8.1 Hz, H-1), 4.65 (d, *J* = 5.5 Hz, H-5), 4.02 (td, *J* = 5.9, 2.8 Hz, H-6), 3.91 (dd, *J* = 6.4, 2.6 Hz, H-9), 3.48–3.32 (m, ethylene bridge CH_2_), 3.26 (dd, *J* = 13.1, 5.3 Hz, H-16), 3.08 (d, *J* = 19.7 Hz, H-10), 2.90 (dd, *J* = 19.9, 6.1 Hz, H-10), 2.83–2.72 (m, H-16, ethylene bridge CH_2_), 2.33 (ddd, *J* = 11.7, 5.3, 2.8 Hz, H-14), 2.01 (td, *J* = 13.6, 4.8 Hz, H-15), 1.83–1.76 (m, H-15), 1.51–1.38 (m, H-7, H-8), 1.00–0.86 (m, H-8); ^13^C-NMR (150 MHz, D_2_O) δ 174.2 (carboxylic C=O), 145.1 (C4), 137.3 (C3), 128.1 (C12), 122.4 (C11), 119.8 (C1), 117.9 (C2), 88.9 (C5), 66.0 (C6), 60.0 (C9), 50.0 (ethylene bridge CH_2_), 46.9 (C16), 40.6 (C13), 38.3 (C14), 34.0 (C15), 29.0 (ethylene bridge CH_2_), 25.9 (C7), 20.5 (C10), 17.6 (C8)

(**27**) *N*-carboxyethyl-norcodeine HCl (3-((7aR)-7-hydroxy-9-methoxy-4,4a,7,7a-tetrahydro-1H-4,12-methanobenzofuro[3,2-e]isoquinolin-3(2H)-yl)propanoic acid hydrochloride) yield: 93%, m.p.: > 200 °C decomp., HR-MS [M + H^+^]: calculated: 358.1649, measured: 358.1634; ^1^H-NMR (600 MHz, D_2_O) δ 6.78 (d, *J* = 8.3 Hz, H-2), 6.65 (d, *J* = 8.3 Hz, H-1), 5.61 (d, *J* = 9.8 Hz, H-7), 5.26 (d, *J* = 9.9 Hz, H-8), 4.94 (dd, *J* = 6.4, 1.3 Hz, H-5), 4.32–4.15 (m, H-6, H-9), 3.71 (s, OCH_3_), 3.51 (q, *J* = 7.1 Hz, ethylene bridge CH_2_), 3.48–3.34 (m, H-16), 3.17 (d, *J* = 20.3 Hz, H-10), 3.00–2.78 (m, H-16, H-14, H-10), 2.75 (td, *J* = 7.0, 2.5 Hz, ethylene bridge CH_2_), 2.22 (d, *J* = 13.9 Hz, H-15), 2.03 (d, *J* = 14.4 Hz, H-15); ^13^C-NMR (150 MHz, D_2_O) δ 175.1 (carboxylic C=O), 146.3 (C4), 142.0 (C3), 133.1 (C7), 129.00 (C12), 125.6 (C8), 124.0 (C11), 120.3 (C1), 114.4 (C2), 90.5 (C5), 65.7 (C6), 58.9 (C9), 56.4 (OCH_3_), 50.6 (ethylene bridge CH_2_), 46.1 (C16), 41.9 (C13), 38.3 (C14), 32.5 (C15), 29.4 (ethylene bridge CH_2_), 21.2 (C10)

(**28**) *N*-carboxyethyl-dihydronorcodeine HCl (3-((7aR)-7-hydroxy-9-methoxy-4,4a,5,6,7,7a-hexahydro-1H-4,12-methanobenzofuro[3,2-e]isoquinolin-3(2H)-yl)propanoic acid hydrochloride) yield: 95%, m.p.: > 220 °C decomp., HR-MS [M + H^+^]: calculated: 360.1805, measured: 360.1791; ^1^H-NMR (600 MHz, D_2_O) δ 6.82 (d, *J* = 8.2 Hz, H-2), 6.70 (d, *J* = 8.2 Hz, H-1), 4.64 (m, H-5), 3.99 (q, *J* = 4.9 Hz, H-6), 3.92 (dd, *J* = 6.3, 2.6 Hz, H-9), 3.73 (s, OCH_3_), 3.43 (dt, *J* = 14.1, 7.2 Hz, ethylene bridge CH_2_), 3.37 (dt, *J* = 13.7, 7.0 Hz, ethylene bridge CH_2_), 3.25 (dd, *J* = 13.2, 4.7 Hz, H-16), 3.10 (d, *J* = 19.8 Hz, H-10), 2.90 (dd, *J* = 19.9, 6.2 Hz, H-10), 2.79 (td, *J* = 7.1, 4.4 Hz, ethylene bridge CH_2_), 2.71 (td, *J* = 13.3, 4.1 Hz, H-16), 2.35 (ddd, *J* = 12.0, 5.4, 2.7 Hz, H-14), 2.03 (td, *J* = 13.7, 4.8 Hz, H-15), 1.77–1.68 (m, H-15), 1.48–1.34 (m, H-8, H-7), 0.90 (tt, *J* = 11.3, 6.0 Hz, H-8); ^13^C-NMR (151 MHz, D_2_O) δ 173.8 (carboxylic C=O), 146.0 (C4), 141.5 (C3), 128.1 (C12), 123.5 (C11), 119.9 (C1), 114.9 (C2), 89.2 (C5), 66.0 (C6), 60.1 (C9), 56.6 (OCH_3_), 49.9 (ethylene bridge CH_2_), 46.9 (C16), 40.5 (C13), 38.1 (C14), 34.1 (C15), 28.9 (ethylene bridge CH_2_), 25.8 (C7), 20.6 (C10), 17.6 (C8)

(**29**) *N*-carboxyethyl-noroxymorphone HCl (3-((7aR)-4a,9-dihydroxy-7-oxo-4,4a,5,6,7,7a-hexahydro-1H-4,12-methanobenzofuro[3,2-e]isoquinolin-3(2H)-yl)propanoic acid hydrochloride) yield: 99%, m.p.: > 280 °C decomp., HR-MS [M + H^+^]: calculated: 360.1442, measured: 360.1332; ^1^H-NMR (600 MHz, D_2_O) δ 6.72 (d, *J* = 8.2 Hz, H-2), 6.69 (d, *J* = 8.3 Hz, H-1), 4.93 (s, H-5), 3.74 (d, *J* = 6.1 Hz, H-9), 3.41 (hept, *J* = 6.8, 5.9 Hz, ethylene bridge CH_2_), 3.34–3.25 (m, H-16, H-10), 3.05 (dd, *J* = 19.9, 6.3 Hz, H-10), 2.89 (td, *J* = 14.8, 5.1 Hz, H-7), 2.85–2.66 (m, H-16, ethylene bridge CH_2_), 2.60 (td, *J* = 13.4, 4.8 Hz, H-15), 2.21 (dt, *J* = 14.8, 3.2 Hz, H-7), 1.95 (ddd, *J* = 14.5, 5.2, 3.0 Hz, H-8), 1.64 (ddd, *J* = 17.9, 13.7, 3.5 Hz, H-8, H-15); ^13^C-NMR (151 MHz, D_2_O) δ 211.5 (C6), 175.0 (carboxylic C=O) 143.2 (C4), 138.7 (C3), 127.2 (C12), 121.7 (C11), 120.9 (C1), 118.7 (C2), 89.5 (C5), 70.6 (C14), 64.0 (C9), 49.7 (ethylene bridge CH_2_), 49.1 (C13), 45.8 (C16), 34.4 (C7), 30.5 (C8), 28.2 (ethylene bridge CH_2_), 27.1 (C15), 23.1 (C10)

(**30**) *N*-carboxyethyl-noroxycodone HCl (3-((7aR)-4a-hydroxy-9-methoxy-7-oxo-4,4a,5,6,7,7a-hexahydro-1H-4,12-methanobenzofuro[3,2-e]isoquinolin-3(2H)-yl)propanoic acid hydrochloride) yield: 96%, m.p.: > 260 °C decomp., HR-MS [M + H^+^]: calculated: 374.1598, measured: 374.1597; ^1^H-NMR (600 MHz, D_2_O) δ 6.87 (d, *J* = 8.3 Hz, H-2), 6.78 (d, *J* = 8.4 Hz, H-1), 4.95 (s, H-5), 3.75 (d, *J* = 4.5 Hz, H-9, OCH_3_), 3.42 (t, *J* = 6.6 Hz, ethylene bridge CH_2_), 3.32 (d, *J* = 20.1 Hz, H-10, H-16), 3.08 (dd, *J* = 20.0, 6.3 Hz, H-10), 2.89 (td, *J* = 14.8, 5.1 Hz, H-7), 2.82 (dt, *J* = 18.2, 6.6 Hz, ethylene bridge CH_2_), 2.70 (td, *J* = 13.5, 12.9, 4.5 Hz, H-16), 2.60 (td, *J* = 13.4, 4.8 Hz, H-15), 2.20 (dt, *J* = 14.9, 3.2 Hz, H-7), 1.95 (ddd, *J* = 14.6, 5.2, 3.0 Hz, H-8), 1.68–1.59 (m, H-8, H-15); ^13^C-NMR (151 MHz, D_2_O) δ 211.0 (C6), 174.9 (carboxylic C=O), 144.3 (C4), 142.6 (C3), 127.1 (C12), 122.4 (C11), 121.1 (C1), 115.6 (C2), 89.4 (C5), 70.2 (C14), 64.0 (C9), 56.7 (OCH_3_), 49.7 (ethylene bridge CH_2_), 48.4 (C13), 45.9 (C16), 34.4 (C7), 30.5 (C8), 28.2 (ethylene bridge CH_2_), 27.2 (C15), 23.1 (C10)

#### 3.2.6. General Synthesis of *N*-Acetylglycine-Nor-Compound Ethyl Esters

The normorphine derivative (2 mmol) was dissolved in 30 mL of acetonitrile. In the presence of sodium bicarbonate (10 mmol) and potassium iodide (2.4 mmol), *N*-(chloroacetyl)glycine ethyl ester (2.4 mmol) was added to the solution. The mixture was stirred and refluxed for 8 h. After the TLC showed no more starting compound, the inorganic salts were filtered and the solvent was evaporated. If it was necessary, column chromatography was used. (chloroform: methanol 9:1)

(**31**) *N*-acetylglycine-normorphine ethyl ester (ethyl 2-(2-((7aR)-7,9-dihydroxy-4,4a,7,7a-tetrahydro-1H-4,12-methanobenzofuro[3,2-e]isoquinolin-3(2H)-yl)acetamido)acetate) yield: 94%, m.p.: 105 °C, HR-MS [M + H^+^]: calculated: 415.1863, measured: 415.1848; ^1^H-NMR (600 MHz, DMSO-*d_6_*) δ 6.42 (d, *J* = 8.0 Hz, H-2), 6.32 (d, *J* = 8.1 Hz, H-1), 5.53 (dp, *J* = 9.9, 1.6 Hz, H-7), 5.20 (dt, *J* = 9.8, 2.8 Hz, H-8), 4.67 (dd, *J* = 6.1, 1.3 Hz, H-5), 4.06 (q, *J* = 7.1 Hz, ester CH_2_), 3.85 (qd, *J* = 17.4, 6.2 Hz, glycine CH_2_), 3.35 (dd, *J* = 6.1, 3.0 Hz, H-9), 3.20 (d, *J* = 16.1 Hz, CH_2_ bridge), 2.98 (d, *J* = 16.1 Hz, CH_2_ bridge), 2.76 (d, *J* = 18.4 Hz, H-10), 2.71 (dd, *J* = 4.7, 2.5 Hz, H-14), 2.55 (dd, *J* = 12.1, 4.6 Hz, H-16), 2.41–2.29 (m, H-10, H-16), 2.05 (td, *J* = 12.5, 4.9 Hz, H-15), 1.61 (d, *J* = 12.7 Hz, H-15), 1.16 (t, *J* = 7.1 Hz, ester CH_3_); ^13^C-NMR (150 MHz, DMSO-*d_6_*) δ 171.0 (amide C=O), 170.3 (ester C=O), 146.7 (C4), 139.1 (C3), 133.8 (C7), 131.2 (C12), 128.7 (C8), 125.4 (C11), 119.0 (C1), 116.7 (C2), 91.8 (C6), 66.9 (C6), 60.8 (ester CH_2_), 58.9 (CH_2_ bridge), 57.6 (C9), 45.0 (C16), 43.5 (C13), 40.9 (glycine CH_2_), 40.7 (C14), 35.7 (C15), 23.3 (C10), 14.5 (ester CH_3_)

(**32**) *N*-acetylglycine-dihydronormorphine ethyl ester (ethyl 2-(2-((7aR)-7,9-dihydroxy-4,4a,5,6,7,7a-hexahydro-1H-4,12-methanobenzofuro[3,2-e]isoquinolin-3(2H)-yl)acetamido)acetate) yield: 91%, m.p.: 76 °C, HR-MS [M + H^+^]: calculated: 417.2020, measured: 417.2020; ^1^H-NMR (600 MHz, DMSO-*d_6_*) δ 6.53 (d, *J* = 8.0 Hz, H-2), 6.42 (d, *J* = 8.0 Hz, H-1), 4.47 (d, *J* = 4.8 Hz, H-5), 4.09 (q, *J* = 7.1 Hz, ester CH_2_), 3.88 (d, *J* = 6.4 Hz, glycine CH_2_), 3.85 (d, *J* = 6.0 Hz, glycine CH_2_), 3.83–3.78 (m, H-6), 3.19 (d, *J* = 16.1 Hz, CH_2_ bridge), 3.07 (d, *J* = 3.2 Hz, H-9), 2.93 (d, *J* = 16.0 Hz, CH_2_ bridge), 2.73 (d, *J* = 18.3 Hz, H-10), 2.48 (m, H-16), 2.38 (t, *J* = 1.8 Hz, H-10), 2.29 (s, H-14), 2.26–2.18 (m, H-16), 1.90 (td, *J* = 12.3, 4.9 Hz, H-15), 1.46 (dt, *J* = 10.8, 2.3 Hz, H-15), 1.40 (dq, *J* = 13.8, 7.1 Hz, H-8), 1.32 (td, *J* = 6.7, 3.6 Hz, H-7), 1.18 (m, ester CH_3_, H-7), 0.86 (d, *J* = 18.8 Hz, H-8); ^13^C-NMR (150 MHz, DMSO-*d_6_*) δ 171.0 (amide C=O), 170.6 (ester C=O), 146.5 (C4), 138.5 (C3), 130.4 (C12), 125.3 (C11), 118.5 (C1), 117.2 (C2), 90.4 (C5), 66.7 (C6), 61.0 (ester CH_2_), 59.2 (CH_2_ bridge), 58.8 (C9), 45.1 (C16), 42.5 (C13), 41.0 (glycine CH_2_), 38.2 (C14), 37.5 (C15), 26.0 (C7), 23.0 (C10), 20.0 (C8), 14.6 (ester CH_3_)

(**33**) *N*-acetylglycine-norcodeine ethyl ester (ethyl 2-(2-((7aR)-7-hydroxy-9-methoxy-4,4a,7,7a-tetrahydro-1H-4,12-methanobenzofuro[3,2-e]isoquinolin-3(2H)-yl)acetamido)acetate) yield: 90%, m.p.: oil, HR-MS [M + H^+^]: calculated: 429.2020, measured: 429.2011; ^1^H-NMR (600 MHz, DMSO-*d_6_*) δ 6.59 (d, *J* = 8.2, H-2), 6.44 (d, *J* = 8.3, H-1), 5.53 (d, *J* = 9.8 Hz, H-7), 5.21 (dt, *J* = 7.4, 3.4 Hz, H-8), 4.69 (d, *J* = 5.4 Hz, H-5), 4.10 (m, H-6), 4.06 (tdd, *J* = 7.2, 4.7, 2.6 Hz, ester CH_2_), 3.86 (m, glycine CH_2_), 3.69 (d, *J* = 3.2 Hz, OCH_3_), 3.37 (dt, *J* = 7.6, 3.8 Hz, H-9), 3.20 (dd, *J* = 16.3, 4.2 Hz, CH_2_ bridge), 2.98 (dd, *J* = 15.7, 3.8 Hz, CH_2_ bridge), 2.80 (dd, *J* = 18.5, 4.1 Hz, H-10), 2.75–2.69 (m, H-14), 2.58 (m, H-16), 2.41–2.31 (m, H-10, H-16), 2.11–1.99 (m, H-15), 1.61 (d, *J* = 12.9 Hz, H-15), 1.16 (ddd, *J* = 7.1, 4.7, 2.3 Hz, ester CH_3_); ^13^C-NMR (150 MHz, DMSO-*d_6_*) δ 170.9 (amide C=O), 170.5 (ester C=O), 147.7 (C4), 141.8 (C3), 133.7 (C7), 131.5 (C12), 128.7 (C8), 127.5 (C11), 118.8 (C1), 113.8 (C2), 92.4 (C5), 67.0 (C6), 60.9 (ester CH_2_), 58.9 (CH_2_ bridge), 55.5 (OCH_3_), 57.7 (C9), 44.9 (C16), 43.6 (C13), 40.9 (glycine CH_2_), 40.6 (C14), 35.7 (C15), 23.4 (C10), 14.5 (ester CH_3_)

(**34**) *N*-acetylglycine-dihydronorcodeine ethyl ester (ethyl 2-(2-((7aR)-7-hydroxy-9-methoxy-4,4a,5,6,7,7a-hexahydro-1H-4,12-methanobenzofuro[3,2-e]isoquinolin-3(2H)-yl)acetamido)acetate) yield: 89% m.p.: 76 °C, HR-MS [M + H^+^]: calculated: 431.2177, measured: 431.2165; ^1^H-NMR (600 MHz, DMSO-*d_6_*) δ 6.70 (d, *J* = 8.2 Hz, H-2), 6.54 (d, *J* = 8.2 Hz, H-1), 4.49 (dd, *J* = 4.9, 3.6 Hz, H-5), 4.08 (q, *J* = 7.1 Hz, ester CH_2_), 3.88 (d, *J* = 6.3 Hz, glycine CH_2_), 3.84 (d, *J* = 5.9 Hz, glycine CH_2_), 3.82 (q, *J* = 3.9 Hz, H-6), 3.76 (s, OCH_3_), 3.20 (d, *J* = 16.1 Hz, CH_2_ bridge), 3.09 (dd, *J* = 6.1, 2.7 Hz, H-9), 2.93 (d, *J* = 16.1 Hz, CH_2_ bridge), 2.77 (d, *J* = 18.4 Hz, H-10), 2.48–2.40 (m, H-10), 2.30 (ddd, *J* = 11.5, 6.7, 2.7 Hz, H-14), 2.20 (td, *J* = 12.2, 3.4 Hz, H-16), 1.90 (td, *J* = 12.4, 4.9 Hz, H-15), 1.45 (dd, *J* = 11.6, 3.1 Hz, H-15), 1.39 (dt, *J* = 13.4, 6.8 Hz, H-8), 1.32 (qd, *J* = 6.8, 4.7 Hz, H-7), 1.18 (t, *J* = 7.1 Hz, ester CH_3_), 0.89 (tt, *J* = 12.7, 6.4 Hz, H-8); ^13^C-NMR (150 MHz, DMSO-*d_6_*) δ 171.0 (amide C=O), 170.4 (ester C=O), 147.4 (C4), 141.3 (C3), 130.6 (C12), 127.2 (C11), 118.5 (C1), 114.5 (C2), 90.7 (C5), 66.4 (C6), 60.8 (ester CH_2_), 59.1 (CH_2_ bridge), 58.6 (C9), 55.9 (OCH_3_), 45.0 (C16), 42.3 (C13), 40.9 (glycine CH_2_), 38.3 (C14), 37.3 (C15), 26.1 (C7), 22.9 (C10), 19.8 (C8), 14.5 (ester CH_3_)

(**35**) *N*-acetylglycine-noroxymorphone ethyl ester (ethyl 2-(2-((7aR)-4a,9-dihydroxy-7-oxo-4,4a,5,6,7,7a-hexahydro-1H-4,12-methanobenzofuro[3,2-e]isoquinolin-3(2H)-yl)acetamido)acetate) yield: 95%, m.p.: 83 °C, HR-MS [M + H^+^]: calculated: 431.1813, measured: 431.1799; ^1^H-NMR (600 MHz, DMSO-*d_6_*) δ 6.56 (d, *J* = 8.1 Hz, H-2), 6.53 (d, *J* = 8.1 Hz, H-1), 4.77 (s, H-5), 4.09 (qd, *J* = 7.1, 1.2 Hz, ester CH_2_), 3.95 (d, *J* = 6.7 Hz, glycine CH_2_), 3.80 (d, *J* = 5.7 Hz, glycine CH_2_), 3.23 (d, *J* = 16.0 Hz, CH_2_ bridge), 3.02–2.89 (m, CH_2_ bridge, H-9, H-7), 2.59–2.53 (m, H-16), 2.53–2.51 (m, H-10), 2.43 (td, *J* = 12.6, 5.0 Hz, H-15), 2.08 (dt, *J* = 14.3, 3.2 Hz, H-7), 1.99 (td, *J* = 12.0, 3.7 Hz, H-16), 1.73 (ddd, *J* = 13.3, 5.2, 3.0 Hz, H-8), 1.29 (dt, *J* = 11.9, 2.7 Hz, H-15), 1.18 (t, *J* = 7.1 Hz, ester CH_3_); ^13^C-NMR (150 MHz, DMSO-*d_6_*) δ 209.3 (C6), 170.5 (amide C=O), 170.4 (ester C=O), 143.8 (C4), 139.8 (C3), 130.1 (C12), 123.9 (C11), 119.5 (C1), 117.6 (C2), 89.7 (C5), 70.7 (C14), 64.0 (C9), 60.9 (ester CH_2_), 59.5 (CH_2_ bridge), 50.1 (C13), 44.2 (C16), 40.9 (glycine CH_2_), 36.2 (C7), 31.6 (C8), 29.8 (C15), 24.5 (C10), 14.5 (ester CH_3_)

(**36**) *N*-acetylglycine-noroxycodone ethyl ester (ethyl 2-(2-((7aR)-4a-hydroxy-9-methoxy-7-oxo-4,4a,5,6,7,7a-hexahydro-1H-4,12-methanobenzofuro[3,2-e]isoquinolin-3(2H)-yl)acetamido)acetate) yield: 96% m.p.: 81 °C, HR-MS [M + H^+^]: calculated: 445.1969, measured: 445.1964; ^1^H-NMR (600 MHz, DMSO-*d_6_*) δ 6.75 (d, *J* = 8.2 Hz, H-2), 6.67 (d, *J* = 8.2 Hz, H-1), 4.85 (s, H-5), 4.09 (qd, *J* = 7.1, 1.4 Hz, ester CH_2_), 3.94 (dd, *J* = 17.3, 6.7 Hz, glycine CH_2_), 3.79 (s, glycine CH_2_), 3.78 (s, OCH_3_), 3.24 (d, *J* = 16.0 Hz, CH_2_ bridge), 3.08–2.92 (m, CH_2_ bridge, H-7, H-10), 2.65–2.54 (m, H-10, H-16), 2.40–2.37 (m, H-15), 2.08 (dt, *J* = 14.1, 3.1 Hz, H-7), 1.97 (td, *J* = 12.0, 3.7 Hz, H-16), 1.75 (ddd, *J* = 13.3, 5.1, 2.9 Hz, H-8), 1.51–1.37 (m, H-8), 1.32–1.27 (m, H-15), 1.19 (t, *J* = 7.1 Hz, ester CH_3_); ^13^C-NMR (150 MHz, DMSO-*d_6_*) δ 209.0 170.4 (amide C=O), 170.3 (ester C=O), (C6), 142.5 (C3), 144.8 (C4), 130.3 (C12), 126.1 (C11), 119.8 (C1), 115.3 (C2), 90.1 (C5), 70.6 (C14), 60.9 (ester CH_2_), 59.4 (CH_2_ bridge), 56.8 (OCH_3_), 50.2 (C13), 44.1 (C16), 40.7 (glycine CH_2_), 36.0 (C7), 31.8 (C8), 63.9 (C9), 29.4 (C15), 24.6 (C10), 14.5 (ester CH_3_)

#### 3.2.7. General Synthesis of *N*-Acetylglycine-Nor-Compounds

The *N*-acetylglycine-nor-compound ethyl ester (0.5 mmol) was dissolved in the mixture of ethanol (1 g) and water (2,5 mL). The mixture was hydrolyzed with sodium hydroxide (1 M, 0.5 mmol) for 1 h at 60 °C. The hydrolysis reaction was monitored by TLC and upon complete disapparance of the starting material, the pH was adjusted to 3–4 with 10% HCl solution. Then the solvent was evaporated to obtain the hydrochloride salt of the desired compound.

(**37**) *N*-acetylglycine-normorphine HCl (2-(2-((7aR)-7,9-dihydroxy-4,4a,7,7a-tetrahydro-1H-4,12-methanobenzofuro[3,2-e]isoquinolin-3(2H)-yl)acetamido)acetic acid hydrochloride) yield: 98%, m.p.: > 205 °C decomp., HR-MS [M + H^+^]: calculated: 387.1551, measured: 387.1547; ^1^H-NMR (600 MHz, D_2_O) δ 6.65 (d, *J* = 8.1 Hz, H-2), 6.57 (d, *J* = 8.2 Hz, H-1), 5.63 (dd, *J* = 9.8, 2.8 Hz, H-7), 5.25 (d, *J* = 10.0 Hz, H-8), 4.96 (dd, *J* = 6.4, 1.2 Hz, H-5), 4.33–4.00 (m, H-6, H-9), 3.94 (s, glycine CH_2_), 3.51 (d, *J* = 7.1 Hz, CH_2_ bridge), 3.38 (s, H-16), 3.15 (d, *J* = 20.1 Hz, H-10), 3.02 (s, H-14), 2.42–2.23 (m, H-15), 2.05 (d, *J* = 14.4 Hz, H-15); ^13^C-NMR (150 MHz, D_2_O) δ 173.1 (carboxylic C=O), 165.3 (amide C=O), 145.5 (C4), 138.0 (C3), 133.1 (C7), 129.2 (C12), 125.5 (C8), 123.0 (C11), 120.3 (C1), 117.7 (C2), 90.3 (C5), 65.2 (C6), 60.4 (C9), 57.6 (CH_2_ bridge), 47.1 (C16), 41.7 (C13), 41.2 (glycine CH_2_), 38.0 (C14), 32.2 (C15), 22.0 (C10)

(**38**) *N*-acetylglycine-dihydronormorphine HCl (2-(2-((7aR)-7,9-dihydroxy-4,4a,5,6,7,7a-hexahydro-1H-4,12-methanobenzofuro[3,2-e]isoquinolin-3(2H)-yl)acetamido)acetic acid hydrochloride) yield: 94%, m.p.: > 225 °C decomp., HR-MS [M + H^+^]: calculated: 389.1707, measured: 389.1765; ^1^H-NMR (600 MHz, D2O) δ 6.83 (d, *J* = 8.3 Hz, H-2), 6.65 (d, *J* = 8.3 Hz, H-1), 4.74 (d, *J* = 5.6 Hz, H-5), 4.41–4.17 (m, H-6, H-9), 4.16–4.00 (m, H-6, H-9), 3.96 (d, *J* = 3.6 Hz, glycine CH_2_) 3.31 (d, *J* = 14.0 Hz, H-16), 3.11 (t, *J* = 19.5 Hz, H-10), 2.89 (d, *J* = 13.5 Hz, CH_2_ bridge), 2.53 (m, H-14), 2.11 (s, H-15), 1.81 (d, *J* = 13.0 Hz, H-15), 1.47 (m, H-8), 0.95 (m, H-8); ^13^C-NMR (150 MHz, D_2_O) δ 172.9 (carboxylic C=O), 165.3 (amide C=O), 145.6 (C4), 138.5 (C3), 128.7 (C12), 123.2 (C11), 120.0 (C1), 117.1 (C2), 89.7 (C5), 66.1 (C6), 61.1 (C9), 57.0 (CH_2_ bridge), 47.8 (C16), 41.4 (glycine CH_2_), 41.2 (C13), 38.2 (C14), 32.9 (C15), 25.7 (C7), 21.6 (C10), 17.9 (C8)

(**39**) *N*-acetylglycine-norcodeine HCl (2-(2-((7aR)-7-hydroxy-9-methoxy-4,4a,7,7a-tetrahydro-1H-4,12-methanobenzofuro[3,2-e]isoquinolin-3(2H)-yl)acetamido)acetic acid hydrochloride) yield: 99%, m.p.: > 200 °C decomp., HR-MS [M + H^+^]: calculated: 401.1707, measured: 401.1704; ^1^H-NMR (600 MHz, D_2_O) δ 6.80 (d, *J* = 8.3 Hz, H-2), 6.66 (d, *J* = 8.3 Hz, H-1), 5.62 (d, *J* = 9.9 Hz, H-7), 5.25 (d, *J* = 10.0 Hz, H-8), 4.97 (d, *J* = 6.3 Hz, H-5), 4.29–4.23 (m, H-6, H-9), 4.16–4.06 (m, CH_2_ bridge, glycine CH_2_), 3.72 (s, OCH_3_), 3.39 (m, H-16), 3.20–3.17 (m, H-10, H-16), 3.03 (m, H-14), 2.33 (t, *J* = 13.0 Hz, H-15), 2.04 (d, *J* = 14.5 Hz, H-15); ^13^C-NMR (150 MHz, D_2_O) δ 173.2 (carboxylic C=O), 165.4 (amide C=O), 146.3 (C4), 142.2 (C3), 133.0 (C7), 128.8 (C12), 125.2 (C8), 123.8 (C11), 120.2 (C1), 114.7 (C2), 90.7 (C5), 65.7 (C6), 60.4 (C9), 56.8 (OCH_3_), 54.5 (CH_2_ bridge), 47.1 (C16), 41.3 (glycine CH_2_), 41.9 (C13), 37.9 (C14), 32.2 (C15), 22.1 (C10)

(**40**) *N*-acetylglycine-dihydronorcodeine HCl (2-(2-((7aR)-7-hydroxy-9-methoxy-4,4a,5,6,7,7a-hexahydro-1H-4,12-methanobenzofuro[3,2-e]isoquinolin-3(2H)-yl)acetamido)acetic acid hydrochloride) yield: 95%, m.p.: > 205 °C decomp., HR-MS [M + H^+^]: calculated: 403.1864, measured: 403.1852; ^1^H-NMR (600 MHz, D_2_O) δ 6.87 (d, *J* = 8.3 Hz, H-2), 6.74 (d, *J* = 8.4 Hz, H-1), 4.70 (d, *J* = 5.4 Hz, H-5), 4.03 (d, *J* = 4.7 Hz, H-6), 3.94 (s, H-9, glycine CH_2_), 3.76 (s, OCH_3_), 3.30 (d, *J* = 13.0 Hz, H-16), 3.12 (d, *J* = 19.4 Hz, H-10), 2.80–2.74 (m, CH_2_ bridge), 2.49 (s, H-14), 2.11 (s, H-15), 1.79 (d, *J* = 14.0 Hz, H-15), 1.48–1.43 (m, H-8), 0.96–0.91 (m, H-8); ^13^C-NMR (150 MHz, D_2_O) δ 172.8 (carboxylic C=O), 165.6 (amide C=O), 145.8 (C4), 141.7 (C3), 127.9 (C12), 123.3 (C11), 120.0 (C1), 115.1 (C2), 89.2 (C5), 66.0 (C6), 61.5 (C9), 57.0 (OCH_3_), 54.4 (CH_2_ bridge), 47.9 (C16), 41.4 (glycine CH_2_), 40.6 (C13), 38.2 (C14), 33.9 (C15), 26.0 (C7), 21.4 (C10), 17.6 (C8)

(**41**) *N*-acetylglycine-noroxymorphone HCl (2-(2-((7aR)-4a,9-dihydroxy-7-oxo-4,4a,5,6,7,7a-hexahydro-1H-4,12-methanobenzofuro[3,2-e]isoquinolin-3(2H)-yl)acetamido)acetic acid hydrochloride) yield: 98%, m.p.: > 190 °C decomp., HR-MS [M + H^+^]: calculated: 403.1500, measured: 403.1495; ^1^H-NMR (600 MHz, D_2_O) δ 6.73 (d, *J* = 8.2 Hz, H-2), 6.70 (d, *J* = 8.3 Hz, H-1), 4.95 (s, H-5), 4.19 (d, *J* = 16.1 Hz, CH_2_ bridge), 3.99–3.88 (m, CH_2_ bridge, glycine CH_2_), 3.83 (d, *J* = 6.1 Hz, H-9), 3.33 (s, H-10), 3.20 (dd, *J* = 13.2, 4.9 Hz, H-16), 3.08 (dd, *J* = 20.1, 6.3 Hz, H-10), 2.90 (td, *J* = 14.8, 4.8 Hz, H-7), 2.68 (td, *J* = 13.5, 5.0 Hz, H-15), 2.22 (dt, *J* = 14.8, 3.2 Hz, H-7), 1.99–1.92 (m, H-8), 1.72–1.59 (m, H-8, H-15); ^13^C-NMR (150 MHz, D_2_O) δ 211.5 (C6), 172.4 (carboxylic C=O), 165.2 (amide C=O), 143.2 (C4), 138.8 (C3), 127.1 (C12), 121.5 (C11), 121.1 (C1), 118.7 (C2), 89.3 (C5), 70.7 (C14), 65.0 (C9), 53.6 (CH_2_ bridge), 48.7 (C13), 47.0 (C16), 41.6 (glycine CH_2_), 34.5 (C7), 30.4 (C8), 27.4 (C15), 23.8 (C10)

(**42**) *N*-acetylglycine-noroxycodone HCl (2-(2-((7aR)-4a-hydroxy-9-methoxy-7-oxo-4,4a,5,6,7,7a-hexahydro-1H-4,12-methanobenzofuro[3,2-e]isoquinolin-3(2H)-yl)acetamido)acetic acid hydrochloride) yield: 93%, m.p.: > 180 °C decomp., HR-MS [M + H^+^]: calculated: 417.1656, measured: 417.1642; ^1^H-NMR (600 MHz, D_2_O) δ 6.89 (d, *J* = 8.3 Hz, H-2), 6.81 (d, *J* = 8.4 Hz, H-1), 4.98 (s, H-5), 4.18 (d, *J* = 16.1 Hz, CH_2_ bridge), 3.95 (d, *J* = 16.2 Hz, CH_2_ bridge), 3.86 (d, *J* = 6.2 Hz, H-9), 3.79 (d, *J* = 3.6 Hz, glycine CH_2_), 3.77 (s, OCH_3_), 3.35 (d, *J* = 20.1 Hz, H-10), 3.22 (dd, *J* = 13.0, 4.8 Hz, H-16), 3.11 (dd, *J* = 20.1, 6.3 Hz, H-10), 2.97–2.83 (m, H-7), 2.69 (td, *J* = 13.5, 5.0 Hz, H-15), 2.22 (dt, *J* = 14.9, 3.3 Hz, H-7), 1.98 (ddd, *J* = 14.5, 5.3, 3.1 Hz, H-8), 1.73–1.66 (m, H-15), 1.63 (td, *J* = 14.6, 3.4 Hz, H-8); ^13^C-NMR (150 MHz, D_2_O) δ 211.3, 175.1 (carboxylic C=O), 165.2 (amide C=O), (C6), 144.0 (C4), 142.8 (C3), 127.1 (C12), 122.5 (C11), 121.0 (C1), 115.6 (C2), 89.4 (C5), 70.8 (C14), 65.0 (C9), 56.5 (OCH_3_), 53.8 (CH_2_ bridge), 48.8 (C13), 47.0 (C16), 42.8 (glycine CH_2_), 34.4 (C7), 30.4 (C8), 27.3 (C15), 23.8 (C10)

### 3.3. NMR-pH Titrations

All NMR measurements were performed on a Varian VNMRS spectrometer (599.9 MHz for ^1^H). Spectra were recorded at 25 °C. Titrations were carried out in solutions containing 90% (*v*/*v*) H_2_O and 10% (*v*/*v*) D_2_O, with the addition of small amounts of 0.1 M HCl and NaOH. An amount of 1 M HCl was also used to achieve highly acidic pH values. The ionic strength was kept at 0.15 M by the presence of NaCl. The concentration of the investigated opioid compounds was 4 mM. The sample volume was 650 μL. NMR spectra were referenced to the internal standard DSS. The water signal was suppressed by presaturation [44]. Spectra were processed using Varian VNMRj 3.2a software (Varian, Inc., NMR Systems, Palo Alto, CA, USA). Initial pH values were read on a Metrohm 2.780.0010 precision pH meter with a 6.0258.600 Unitrode glass Pt 1000 electrode (Metrohm AG, Herisau, Switzerland). The exact pH in the NMR tube was measured by NMR-pH indicator molecules chloroacetic acid and dichloroacetic acid [45] to avoid the uncertainty of the glass electrode pH-meter readings in highly acidic solutions. The concentration of these indicators was 1.0 mM. For the analysis of NMR titration curves of proton chemical shifts versus pH, the software Origin Pro 8 (OriginLab Corp., Northampton, MA, USA) was used [46].

### 3.4. pH-Potentiometric Titrations

A 716 DMS Titrino automatic titrator (Metrohm) (Metrohm AG, Herisau, Switzerland) with a Metrohm 6.0234.110 combined pH glass electrode was used for the potentiometric titrations, under automatic PC (personal computer) control. The pH-potentiometric system was calibrated using pH 1.68, 4.01, 6.87, 9.18 aqueous National Bureau of Standards (NBS) buffer solutions. Constant temperature (25 ± 0.1 °C) was provided by a thermostated double-walled glass cell. Difference titrations were carried out in the absence (blank) and presence of ligands. First 2.0 mL of 0.1 M HCl solutions were titrated with 0.1 M KOH. Constant ionic strength of 0.15 M was provided by the presence of KCl. Then a ligand was added to the same volume of HCl solution and was subsequently titrated with KOH. The initial concentration of the ligands varied between 1.5 and 4.0 mM in the titrations, and at least three parallel measurements were carried out for each ligand. Non-Linear parameter fitting with Origin 8 provided the protonation constants from the volume differences [47].

## 4. Conclusions

Two reaction ways have been successfully developed to obtain nitrogen linked free carboxylic group containing morphine-derivative haptens, utilizing ethyl bromoacetate and ethyl acrylate, followed by alkaline hydrolysis. Although the peptide bond formation was not successful, an alternative synthesis route that used *N*-acetylglycine ethyl ester led to the desired glycine connected hapten derivatives. As a result, 36 compounds were synthesized and only two of them were previously known. Detailed NMR and HR-MS measurements confirmed the structure of all novel molecules.

In the *N*-acetylglycine opioid compounds, the basicity of the phenolate site is much larger than that of the amino site because of the electron withdrawing amide group. Hydrogenation of the C7-C8 double bond, similarly as the replacement of the electron withdrawing C6 keto group by a hydroxyl group, increases the electron density and thus the basicity of both sites. In the *N*-carboxyalkyl opioid compounds the basicity of the amino and phenolate site is within an order of magnitude in each tribasic molecule. The higher amino basicity of the carboxylic acids compared to their ester derivatives can be explained by the presence of a negatively charged carboxylate group in the pH range of the amino protonation. On the other hand, the protonation state of the carboxylate group does not significantly influence the basicity of the far-away phenolate site. The carboxylate group is less basic in the *N*-carboxymethyl than in the *N*-carboxyethyl derivatives, because the protonated, electron withdrawing amino group is closer to the carboxylate in the former class of compounds.

Some of these compounds are screened for opioid activity. These studies are in progress. As continuation of this project, changing the length of the linker and coupling of other amino acids, is planned.

## Figures and Tables

**Table 1:** Protonation constants of the *N*-acetylglycine opioid compounds. The standard deviations are in brackets.

Compounds	log*K*_1_	log*K*_2_	log*K*_3_
*N*-acetylglycine-normorphine (**37**)	9.27 (0.03)	6.06 (0.01)	3.18 (0.02)
*N*-acetylglycine-normorphine ethyl ester (**31**)	9.19 (0.05)	5.53 (0.04)	-
*N*-acetylglycine-norcodeine (**39**)	5.87 (0.04)	3.18 (0.01)	-
*N*-acetylglycine-norcodeine ethyl ester (**33**)	5.53 (0.05)	-	-
*N*-acetylglycine-dihydronormorphine ethyl ester (**32**)	9.33 (0.04)	6.30 (0.03)	-
*N*-acetylglycine-dihydronorcodeine (**40**)	6.68 (0.05)	3.20 (0.02)	-
*N*-acetylglycine-dihydronorcodeine ethyl ester (**34**)	6.21 (0.04)	-	-
*N*-acetylglycine-noroxymorphone (**41**)	9.08 (0.05)	5.94 (0.04)	3.17 (0.02)
*N*-acetylglycine-noroxymorphone ethyl ester (**35**)	8.95 (0.05)	5.44 (0.02)	-
*N*-acetylglycine-noroxycodone (**42**)	5.83 (0.05)	3.19 (0.02)	-
*N*-acetylglycine-noroxycodone ethyl ester (**36**)	5.32 (0.03)	-	-

**Table 2:** Protonation macroconstants of the *N*-carboxyalkyl opioid compounds. The standard deviations are in brackets.

Compounds	log*K*_1_	log*K*_2_	log*K*_3_
*N*-carboxyethyl-normorphine (**25**)	9.54 (0.05)	8.17 (0.06)	3.27 (0.06)
*N*-carboxyethyl-normorphine ethyl ester (**13**)	9.33 (0.08)	7.34 (0.01)	
*N*-carboxyethyl-norcodeine (**27**)	8.38 (0.03)	3.27 (0.04)	
*N*-carboxyethyl-norcodeine ethyl ester (**15**)	7.34 (0.03)		
*N*-carboxyethyl-dihydronormorphine (**26**)	9.66 (0.06)	8.51 (0.08)	3.23 (0.06)
*N*-carboxyethyl-dihydronormorphine ethyl ester (**14**)	9.48 (0.06)	7.89 (0.03)	
*N*-carboxyethyl-dihydronorcodeine (**28**)	8.76 (0.03)	3.23 (0.06)	
*N*-carboxyethyl-dihydronorcodeine ethyl ester (**16**)	8.00 (0.04)		
*N*-carboxyethyl-noroxymorphone (**29**)	9.66 (0.03)	8.48 (0.01)	3.00 (0.07)
*N*-carboxyethyl-noroxymorphone ethyl ester (**17**)	9.15 (0.01)	7.29 (0.02)	
*N*-carboxyethyl-noroxycodone (**30**)	8.75 (0.06)	2.99 (0.04)	
*N*-carboxyethyl-noroxycodone ethyl ester (**18**)	7.29 (0.03)		
*N*-carboxymethyl-normorphine (**19**)	9.38 (0.08)	8.08 (0.03)	1.87 (0.01)
*N*-carboxymethyl-normorphine ethyl ester (**7**)	9.15 (0.08)	5.56 (0.03)	
*N*-carboxymethyl-norcodeine (**21**)	8.33 (0.07)	1.87 (0.01)	
*N*-carboxymethyl-norcodeine ethyl ester (**9**)	5.64 (0.04)		
*N*-carboxymethyl-dihydronormorphine (**20**)	9.75 (0.08)	8.53 (0.08)	1.90 (0.01)
*N*-carboxymethyl-dihydronormorphine ethyl ester (**8**)	9.22 (0.04)	6.33 (0.01)	
*N*-carboxymethyl-dihydronorcodeine (**22**)	8.86 (0.02)	1.88 (0.01)	
*N*-carboxymethyl-dihydronorcodeine ethyl ester (**10**)	6.33 (0.02)		
*N*-carboxymethyl-noroxymorphone (**23**)	9.28 (0.08)	7.98 (0.03)	1.74 (0.01)
*N*-carboxymethyl-noroxymorphone ethyl ester (**11**)	9.05 (0.03)	5.81 (0.08)	
*N*-carboxymethyl-noroxycodone (**24**)	8.07 (0.06)	1.70 (0.01)	
*N*-carboxymethyl-noroxycodone ethyl ester (**12**)	5.81 (0.04)		

**Table 3:** Protonation microconstants and pair-interactivity parameters of the investigated dibasic compounds.

**Ethers**	**(27)**	**(28)**	**(30)**	**(21)**	**(22)**	**(24)**
$\log k^{N}$	8.38	8.76	8.75	8.33	8.86	8.07
$\log k^{C}$	4.31	3.99	4.45	4.56	4.41	3.90
$\log k_{C}^{N}$	7.34	8.00	7.29	5.64	6.33	5.81
$\log k_{N}^{C}$	3.27	3.23	2.99	1.87	1.88	1.70
$\log E_{C,N}$	1.04	0.76	1.46	2.69	2.53	2.26
**Esters**	**(13)**	**(14)**	**(17)**	**(7)**	**(8)**	**(11)**
$\log k^{O}$	9.33	9.37	9.15	9.07	9.22	9.05
$\log k^{N}$	8.08	8.54	7.87	6.33	6.95	6.29
$\log k_{N}^{O}$	8.59	8.83	8.57	8.38	8.60	8.57
$\log k_{O}^{N}$	7.34	8.00	7.29	5.64	6.33	5.81

**Table 4:** Protonation microconstants and pair-interactivity parameters of the investigated dibasic compounds.

Compounds	(25)	(26)	(29)	(19)	(20)	(23)
Phenolate Microconstants						
$\log k^{O}$	9.33	9.41	9.39	9.13	9.42	9.19
$\log k_{N}^{O}$	8.59	8.87	8.81	8.44	8.80	8.71
$\log k_{C}^{O}$	9.33	9.37	9.15	9.07	9.22	9.05
$\log k_{C,N}^{O}$	8.59	8.83	8.57	8.38	8.60	8.57
Amino Microconstants						
$\log k^{N}$	9.12	9.30	9.33	9.02	9.48	8.55
$\log k_{O}^{N}$	8.38	8.76	8.75	8.33	8.86	8.07
$\log k_{C}^{N}$	8.08	8.54	7.87	6.33	6.95	6.29
$\log k_{C,O}^{N}$	7.34	8.00	7.29	5.64	6.33	5.81
Carboxylate Microconstants						
$\log k^{C}$	4.31	4.03	4.70	4.62	4.63	4.14
$\log k_{N}^{C}$	3.27	3.27	3.24	1.93	2.10	1.88
$\log k_{O}^{C}$	4.31	3.99	4.46	4.56	4.43	4.00
$\log k_{N,O}^{C}$	3.27	3.23	3.00	1.87	1.90	1.74
Pair-Interactivity Parameters						
$\log E_{C,N}$	1.04	0.76	1.46	2.69	2.53	2.26
$\log E_{N,O}$	0.74	0.54	0.58	0.69	0.62	0.48
$\log E_{C,O}$	0.00	0.04	0.24	0.06	0.20	0.14
